# Structure and function of cohesin’s Scc3/SA regulatory subunit

**DOI:** 10.1016/j.febslet.2014.08.015

**Published:** 2014-10-16

**Authors:** Maurici B. Roig, Jan Löwe, Kok-Lung Chan, Frédéric Beckouët, Jean Metson, Kim Nasmyth

**Affiliations:** a Department of Biochemistry, University of Oxford, Oxford OX1 3QU, United Kingdom; b MRC Laboratory of Molecular Biology, Structural Studies Division, Francis Crick Avenue, Cambridge CB2 0QH, United Kingdom

**Keywords:** Scc3, SA/STAG domain, Eco1 acetylation, Releasing activity, Cohesin complex, Sister chromatid separation, Maintenance of cohesion, Smc proteins

## Abstract

Sister chromatid cohesion involves entrapment of sister DNAs by a cohesin ring created through association of a kleisin subunit (Scc1) with ATPase heads of Smc1/Smc3 heterodimers. Cohesin’s association with chromatin involves subunits recruited by Scc1: Wapl, Pds5, and Scc3/SA, in addition to Scc2/4 loading complex. Unlike Pds5, Wapl, and Scc2/4, Scc3s are encoded by all eukaryotic genomes. Here, a crystal structure of Scc3 reveals a hook-shaped protein composed of tandem α helices. Its N-terminal domain contains a conserved and essential surface (CES) present even in organisms lacking Pds5, Wapl, and Scc2/4, while its C-terminal domain binds a section of the kleisin Scc1. Scc3 turns over in G2/M while maintaining cohesin’s association with chromosomes and it promotes de-acetylation of Smc3 upon Scc1 cleavage.

## Highlights

• Crystal structure of cohesin subunit Scc3/SA, showing irregular HEAT-like repeats.
• Scc3 C-terminal domain binds Scc1, cohesin’s kleisin.
• Scc1’s Scc3 binding region mapped.
• Scc3 turns over in G2/M while maintaining cohesin’s association with chromosomes.
• Scc3 promotes de-acetylation of Smc3 upon Scc1 cleavage.

## 1 Main text

Elucidation by X-ray crystallography of the structures of the interfaces between cohesin’s Smc1, Smc3, and kleisin subunits has been crucial in supporting the idea that they form a hetero-trimeric ring capable of entrapping the sister DNAs of small circular mini-chromosomes [10], [13], [14], [19]. Despite being important for demonstrating DNA entrapment and addressing how DNAs enter and exit cohesin rings, knowledge concerning the structure of Smc/kleisin interfaces is insufficient for understanding cohesin dynamics in vivo. Cohesin’s association with and dissociation from chromosomes as well as its acetylation by Eco1 during S phase depends on regulatory subunits such as Wapl, Pds5 [27], and Scc3 [20], [37] that bind the ring via its kleisin subunit as well as a separate Scc2/4 loading complex [6]. Structural information exists for only one of these, namely a non-essential subunit called Wapl, whose conserved C-terminal domain consists of eight HEAT repeats [5], [26].

## 2 The structure of Scc3

**Fig. 1:**
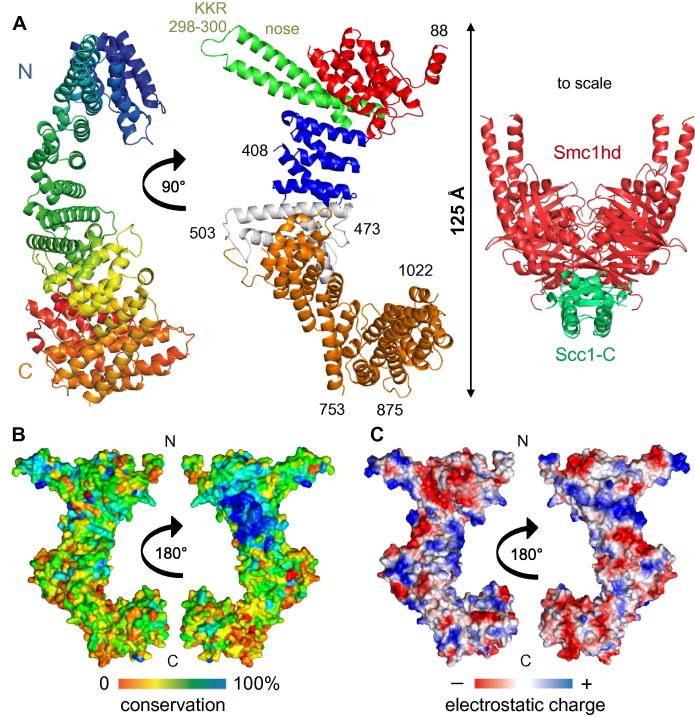
Crystal structure of *Z. rouxii* Scc3 at 2.6 Å resolution. (A) The structure of the *Zr Scc3* fragment (residues 88–1022) reveals an N-terminal end with 9 irregularly distributed α-helices (residues 88–255, in red, middle), followed by a long and partially protruding helix-loop-helix (residues 256–340, in green) and 3 HEAT repeats (residues 341–450, in blue). The C-terminal half of the protein is composed of a continuously twisted tandem array of 8 anti-parallel α-helices resembling tandem HEAT repeats (residues 558–1022, in orange). The N- and C-terminal halves are linked by 4 inter-crossed α-helices (residues 451–557, in grey) that mediate an orthogonal change of the axis between the two halves of the protein. The structure of an Smc1 ATPase head dimer bound to Scc1’s C-terminal winged helix [14] is shown at the same scale, to emphasize Scc3’s size (right). (B) Surface conservation of Scc3 orthologs projected on the surface of *Zr Scc3* shows a clear patch of conservation on one face of the N-terminal half of the protein, largely confined to the surface of the 3 canonical HEAT repeats and the base of the protruding helix-loop-helix. For the multiple alignment conservation, the following sequences were included: *Zygosaccharomyces rouxii* (C5DWM3), *Saccharomyces cerevisiae* (P40541), *Ashbya gossypii* (M9MYD6), *Homo sapiens* (Q6P275), *Xenopus laevis* (Q9DGN1), *Danio rerio* (B0V0X2), *Drosophila melanogaster* (Q9VM62), *Daphnia pulex* (E9FY68), *Brugia malayi* (A8QED2), *Vitis vinifera* (D7TP60), *Candida albicans* (C4YFQ5), *Schizosaccharomyces pombe* (O13816), *Sordaria macrospora* (F7W0E2), *Dictyostelium purpureum* (F0Z8J2). (C) Calculated electrostatic potential of *Zr Scc3* (PyMOL).

Because all eukaryotic genomes encode orthologs, Scc3 is the most important of cohesin’s regulatory subunits. It has roles in loading of cohesin onto chromosomes [16], [22] as well as its subsequent release [15], [30]. Given this functional complexity, any further dissection of Scc3’s functions will depend on knowledge about its molecular architecture. To this end, we purified following expression in *Escherichia* *coli* a version of *Saccharomyces cerevisiae* (*Sc*) Scc3 lacking its unstructured N- and C-terminal extensions. However, this only yielded poorly diffracting crystals with a large unit cell. Trypsin digestion produced a smaller 45 kDa C-terminal domain (*Sc Scc3*–9; residues 674–1072) that yielded excellent crystals whose structure was solved at 2.1 Å (Fig. 1-fig.sup.1B, Table 1), revealing an irregular crescent-shaped helical repeat protein. To obtain the structure of a more complete version of the protein, we expressed Scc3 trimmed of its N- and C-terminal extensions from other yeast species. Of these, Scc3 containing residues 88–1035 from *Zygosaccharomyces rouxii* (*Zr Scc3*) yielded crystals suitable for crystallography. Diffraction data were obtained to a resolution of 2.6 Å from crystals grown at pH 6.5 in a PEG/KSCN condition and their structure solved by selenomethionine single-wavelength anomalous dispersion (SAD, Table 1). Crucially, the equivalent fragment of the *S. cerevisiae* ortholog (120–1060) was capable of supporting proliferation, implying that the *Z. rouxii* Scc3 protein crystallized contains all key functional domains (Fig. 1-fig.sup.2A and B). Furthermore, the equivalent C-terminal domains from *S. cerevisiae* and *Z. rouxii* (Fig. 1-fig.sup.1A and B) had nearly identical structures, confirming the validity of our approach of using a close homolog of *S. cerevisiae* Scc3 for structural studies.

**Table 1:** Crystallographic data.

	*Sc Scc3*–*9* SeMet	*Sc Scc3*–*9* Native	*Zr Scc3* SeMet	*Zr Scc3* Native
Components	*S. cerevisiae* Scc3M-674-1072-HHHHHH	*S. cerevisiae* Scc3M-674-1072-HHHHHH	*Z. rouxii* Scc3M-88-1035-HHHHHH	*Z. rouxii* Scc3M-88-1035-HHHHHH
UniProt/NCBI ID	SCC3_YEAST	SCC3_YEAST	XP_002497125.1	XP_002497125.1

*Data collection*				
Beamline	ESRF id23eh1	ESRF id23eh1	Diamond I04-1	Diamond I04
Wavelength (Å)	0.97940	0.97940	0.91730	1.0332

*Crystal*				
Space group	P1	P1	P2_1_2_1_2_1_	P2_1_2_1_2_1_
Cell (Å)	56.7, 57.8, 79.8 80.7°, 81.9°, 63.8°	56.8, 58.0, 80.2, 80.7°, 82.0°, 64.0°	73.3, 109.7, 159.0	73.4, 109.2, 159.0

*Scaling*				
Resolution (Å)	3.0	2.1	3.0	2.6
Number of crystals	1	1	4	1
Completeness (%)^a^	87.4 (48.5)	97.5 (96.6)	99.9 (99.9)	99.7 (99.7)
Multiplicity^a^	3.8 (3.7)	1.9 (1.9)	40.3 (39.8)	3.7 (3.7)
Ano completeness (%)^a^	83.6 (43.9)		99.7 (97.7)	
Ano multiplicity^a^	1.9 (1.9)		21.2 (20.5)	
Ano correlation^a^^,^^b^	0.447 (0.195)		0.124 (0.089)	
*I*/*σI*^a^	17.1 (10.2)	5.0 (1.8)	21.1 (5.9)	14.3 (3.5)
*R*_pim_^a^	0.050 (0.095)	0.124 (0.406)	0.055 (0.211)	0.045 (0.236)
CC1/2^a^^,^^b^	0.996 (0.985)	0.998 (0.825)	0.997 (0.930)	0.997 (0.836)

*Phasing*				
Scatterer/mode	SeMet	MR from SeMet	SeMet	MR from SeMet
Number of sites	15		8	

*Refinement*				
Model		Dimer of 674–692, 698–710, 720–880, 889–1059; 401 H_2_O		Monomer of 88–225, 235–397, 408–581, 605–753, 761–838, 849–1022; 209 H_2_O
*R*/*R*_free_^c^		0.187 (0.245)		0.187 (0.245)
Bond length rmsd (Å)		0.016		0.015
Bond angle rmsd (°)		1.872		1.549
Favoured (%)^d^		99.7		99.8
Disallowed (%)^d^		0.0		0.0
PDB ID		4UVJ		4UVK

a Values in parentheses refer to the highest recorded resolution shell.

b Correlation coefficient between half sets (CCP4 SCALA).

c 5% of reflections were randomly selected before any refinement.

d Percentage of residues in Ramachandran plot areas (CCP4 PROCHECK).

The crystal structure of *Zr* Scc3 (Fig. 1A) reveals a long, partly twisted and partly crescent-shaped protein composed entirely of α-helices stacked on each other in a surprisingly irregular manner, given the repeat nature of the protein. Because of the helical repeat nature of the protein, the polypeptide chain runs from one end of the molecule to the other, zigzagging along the entire structure (Fig. 1A left). The stacks of anti-parallel α-helices, some of which contain the signature residues Asp19/Arg25 frequently found in HEAT repeats (e.g. D341/R347, D380/R386, and D428/R434), run orthogonal to the main axis of the protein. In addition to a pronounced hook within the protein’s C-terminal half, a striking feature is a “nose” at the N-terminal end, a morphology generated by a pair of anti-parallel helices that are about twice as long as their neighbors. At the tip of the nose reside three conserved basic residues, KKR (298–300). Because sequence homology among Scc3 orthologs stretches throughout the entire *Z. rouxii* structure (Fig. 1-fig.sup.3), most if not all features are likely to be shared by orthologs from a wide variety of eukaryotes including most animals, plants, and protozoa. It is nevertheless striking that a sizeable but clearly defined patch on one surface of three tandem HEAT repeats is much more conserved than all others, a region close to but not enclosing the N-terminal nose (Figs. 1B, 5A, and Fig. 1-fig.sup.3). Though part of this conserved and essential surface (‘CES’) is positively charged (Fig. 1C), it also contains numerous conserved aromatic residues, namely *Zr* H337, F339, Y373, W376, and F417. The highly conserved positively charged residues *Zr* K340, K372, and R416 all protrude from the surface in a conspicuous manner. It is also striking that the helical stack is less twisted and thinner in this region, with the result that the CES presents a relatively flat surface. Scc3 is a large protein whose longest dimension stretches 125 Å, a size that dwarfs the Smc1 ATPase head bound to the C-terminal domain of Scc1 (Fig. 1A, right), to which it binds.

**Fig. 2:**
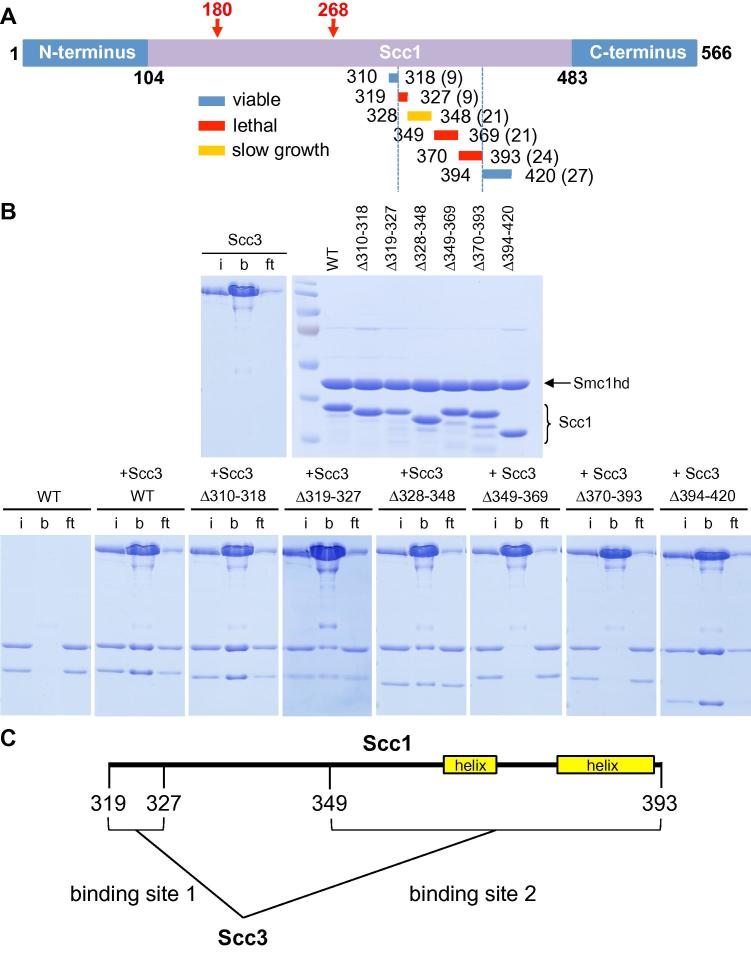
*Sc Scc3* binds to two essential regions of *Sc* Scc1. (A) Representation of *Saccharomyces cerevisiae* Scc1 with systematic deletions between residues 310 and 420 (viable deletions are in blue, lethal in red, slow growth phenotype in yellow) (K16524, K16525, K16526, K17128). (B) Co-purified Smc1 ATPase heads with an Scc1 fragment containing the half C-terminal end (residues 310–566) with the indicated Scc1 deletions were incubated and co-immunoprecipitated with Flag-tagged Scc3. Samples were subjected to SDS–PAGE and Coomassie staining (i: input, b: bound, ft: flow through). Scc1 deletions with a lethal phenotype in (A) show a decrease in Scc3 binding. (C) Schematic representation to scale of the two Scc3 binding sites within Scc1 and the position of a predicted α-helix (yellow) by PSIPRED. The Scc1 regions required for cell viability and Scc3 binding lie on an unstructured region of Scc1 (residues 319–327) and on a region containing 2 α-helices (residues 349–393).

**Fig. 3:**
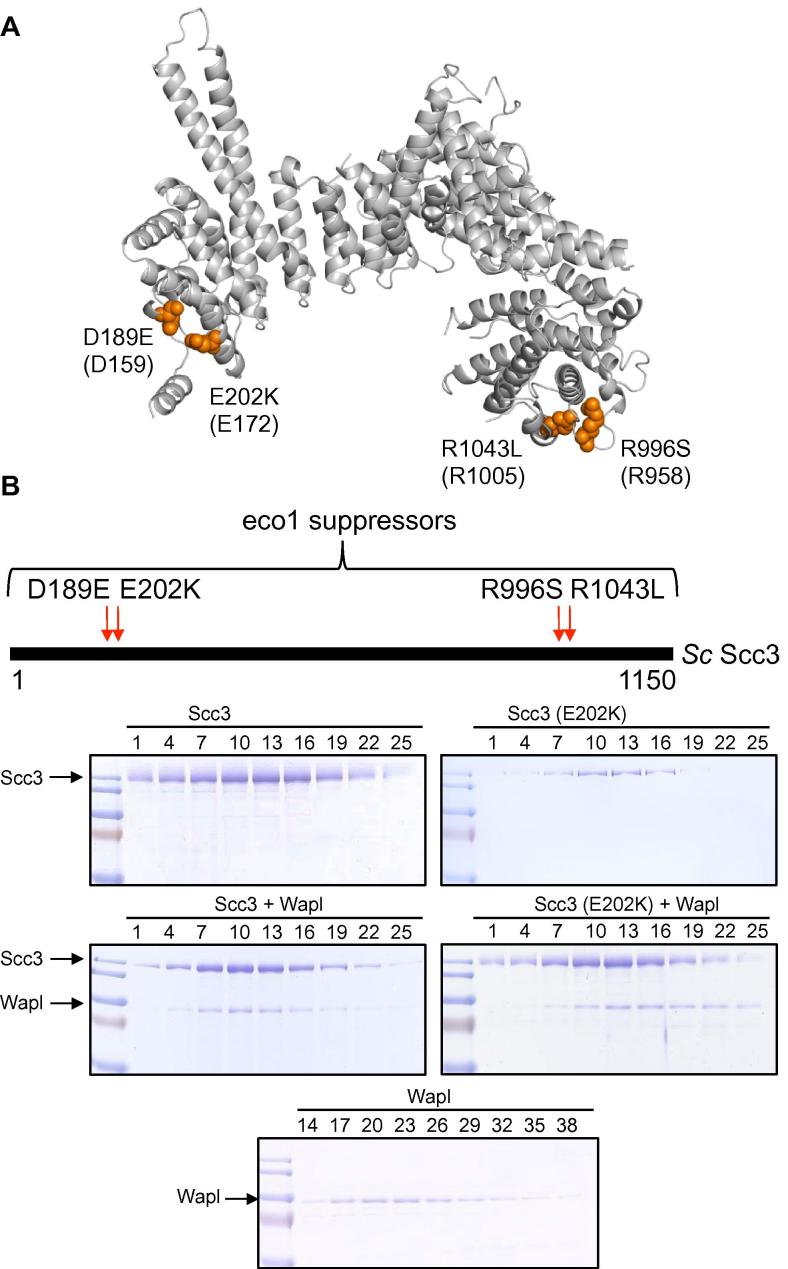
*Sc Scc3* releasing activity mutants. (A) *Sc Scc3* mutations that suppress lethality due to lack of Eco1, namely D189E, E202K, R996S and R1043L [30], are mapped on the *Zr* Scc3 structure (*Zr* Scc3 nomenclature in brackets). Scc3E202K has been shown by photo-bleaching experiments to reduce cohesin’s turnover within centromeres [4]. D189E and E202K are surface residues on α-helices facing each other and closing a pocket-like structure at the N-terminal end. At the other end of the protein R996S and R1043L are exposed residues on a flexible loop and a proximal α-helix, respectively. (B) Binding of *Sc* Scc3 (E202K) to *Sc* Wapl. Scc3 wild type or the mutant E202K were incubated either alone or with Wapl. After separation of the proteins by gel filtration, fractions depicted above each lane were analyzed by SDS–PAGE and Coomassie staining.

**Fig. 4:**
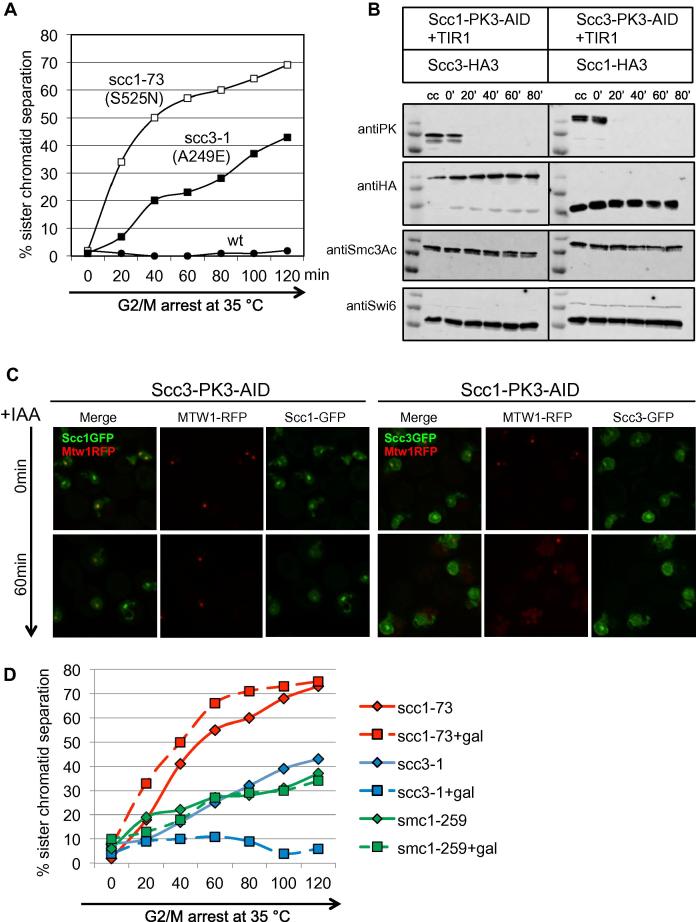
*Sc* Scc3 is required to maintain cohesion. (A) Percentage of sister chromatid separation measured by counting the fraction of cells with double (split) GFP dots at the *URA3* locus in wild type (K15024) or temperature sensitive *scc1*–*73* S525N (K15031) and *scc3*–*1* A249E (K15071) mutants. Cells growing at the permissive temperature (25 °C) were first arrested in G1 by α factor and then released into YEPD medium containing methionine, which represses Cdc20 expression. Following replication (80 min after release) cultures were shifted to the non-permissive (35 °C) (*T* = 0 min). FACS profiles are shown in Fig. 4-fig.sup.2A. (B) Cells with endogenous Scc1 (K20787) or Scc3 (K20783) tagged at their C-termini with the auxin-degron (AID) were uniformly arrested in G2/M following growth in the presence of nocodazole for 120 min at 25 °C. Auxin (5 mM) was then added (*T* = 0 min) to induce protein degradation. Scc1, Scc3 and acetylated Smc3 levels were measured at the indicated time points using Western blotting. (C) Live cell imaging showed peri-centric Scc1 or Scc3 tagged at their C-termini with GFP in G2/M cells arrested in nocodazole (*T* = 0 min). Degradation of Scc3 (K20854) or Scc1 (K23181) by the auxin-inducible degron caused a loss of peri-centric cohesin structures and an increase of GFP signals in the nucleoplasm after 20 min when observing Scc1-GFP or Scc3-GFP respectively, while peri-centric cohesin is unaltered in the absence of the ligase TIR1 (K23382, K23385). Centromeres were marked by Mtw1-RFP only on the cells expressing the TIR ligase (KN20854, KN23181), but not on the control cells (minus TIR) (KN23382, KN23385). Both populations (minus/plus TIR) where mixed for each respective degron for a further quantification of peri-centric cohesin on the same slide (Fig. 4-fig.sup 3). (D) The percentage of cells with double (split) GFP dots measured in temperature sensitive strains *scc1*–*73* (K16680), *scc3*–*1* (K16678) and *smc1*–*259* (K16679) cells in which the wild type versions of these genes under control of the *GAL1*–*10* promoter were induced by addition of galactose following prior arrest in metaphase as described in (A). Cells were shifted to the restrictive temperature (35 °C) 30 min after galactose addition (*T* = 0). FACS profiles and Western blots showing induction of Scc1, Smc1, and Scc3 proteins shown in Fig. 4-fig.sup.4A and B.

**Fig. 5:**
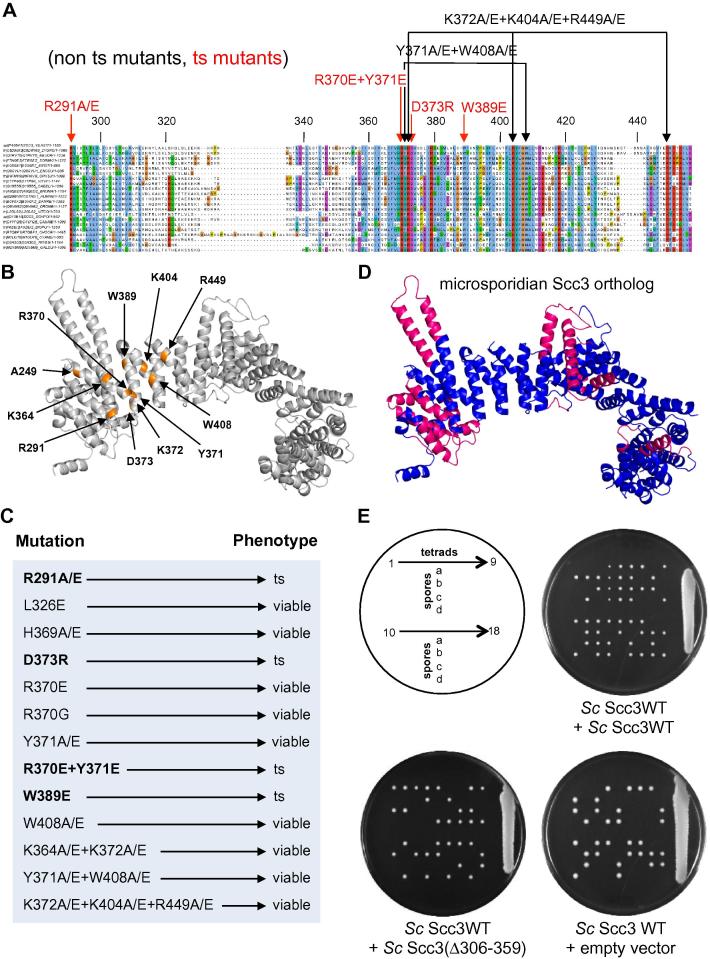
Mutagenesis of *Sc* Scc3. (A) A multiple sequence alignment (ClustalW) of evolutionary divergent eukaryotic organisms, including microsporidia and red algae, shows the degree of homology on the stromal antigen domain (SA/STAG) characteristic of Scc3 orthologues. The following sequences were included in the alignment: *Saccharomyces cerevisiae* (P40541), *Zygosaccharomyces rouxii* (C5DWM3), *Neurospora crassa* (Q7RVT5), *Sordaria macrospora* (F7W0E2), *Encephalitozoon intestinalis* (E0S6N7), *Encephalitozoon cuniculi* (Q8SVU1), *Oryza sativa subsp. Japonica* (B9FMV9), *Vitis vinifera* (D7TP60), *Caenorhabditis elegans* (Q19555), *Brugia malayi* (A8QED2), *Homo sapiens* (Q8WVM7), *Danio rerio* (B0V0X2), *Drosophila melanogaster* (Q9VM62), *Vittaforma corneae* (L2GL62), *Schizosaccharomyces pombe* (O13816), *Candida albicans* (C4YFQ5), *Dictyostelium purpureum* (F0Z8J2), *Chondrus crispus* (R7QBF1), *Cyanidioschyzon merolae* (M1UUT8), *Trypanosoma brucei gambiense* (D0A303), *Galdieria sulphuraria* (M2X5M9). (B) Position within the *Zr* Scc3 structure of highly conserved residues mutagenized within *Sc* Scc3, including *scc3*–*1* (A249E) [37]. (C) Summary of mutant phenotypes. Growth of cells on YEPD plates at 25, 30, and 37 °C shown in Fig. 5-fig.sup.1. (D) Marked in pink are α helices missing in a variety of microsporidian Scc3 proteins (sequence alignments shown in Fig. 5-fig.sup.3). (E) Schematic representation of tetrad dissection and spore position on YPD plates from heterozygous diploids Scc3/scc3Δ. The diploid cells carrying one copy of the endogenous Scc3 gene were transformed to express an additional allele of Scc3 ectopically integrated (either wild type, mutant Δ306–359, or an empty vector). Transformed diploids were sporulated and tetrads were dissected to analyze the phenotype of the resulting haploid cells. Haploid cells expressing only the endogenous Scc3 or the wild type allele of Scc3 ectopically integrated (K21815) are viable, showing more than two viable spores per tetrad, whereas haploid cells expressing only the mutant (Δ306–359) allele lacking Scc3’s nose (K23306), or an empty vector (K21777) are lethal, showing no more than two viable spores per tetrad. Further marker selection and genotype sequencing confirmed that Δ306–359 causes lethality.

Somewhat surprisingly, the Protein Data Bank (PDB) contains no close structural homologs given that HEAT repeat-containing proteins are very common, although it needs to be mentioned that HEAT repeat proteins in general show great variety in structure and repeat architecture. Similarities exist in stretches that adhere most closely to the HEAT repeat theme but the overall architecture has no known equivalent. For example, Scc3’s C-terminal domain resembles parts of the nucleoporin Nup188 (PDB 4KF8). Both proteins share similar helical repeats and connectivity (Fig. 1-fig.sup.4A) but differ in the length, angles and bending of their helices; hence a high RMSD of ∼4 Å results when both proteins are superimposed. Another example from a long list of possible partial structural alignments is transportin 1 (PDB 2H4M, Fig. 1-fig.sup.4B), another helical repeat protein, which aligns poorly with the central part of Scc3 with a high RMSD.

## 3 Scc3’s C-terminal domain binds to two small sections within Scc1

To map which part of *S. cerevisiae* Scc1 binds Scc3, we co-expressed an Smc1 ATPase head domain with the C-terminal half of Scc1 bearing a variety of small deletions and tested the ability of the various Smc1–Scc1 complexes to bind *S. cerevisiae* Scc3 (Fig. 2B). This analysis revealed two regions required for binding, namely *Sc*Scc1K319-Q327 and Scc1S349-K393, both of which are essential for Scc1 function in vivo (Fig. 2A–C). A fragment containing this part of Scc1 bound as well to the C-terminal *Sc Scc3*–9 fragment as to full-length protein (Fig. 2-fig.sup.1A), implying that this part of Scc3 is largely responsible for binding Scc1. We found no evidence of an essential Scc3 binding site within the N-terminal half of Scc1 [44], although we presently cannot exclude non-essential binding sites. Despite many attempts, we were unable to obtain co-crystals suitable for structure determination, with *S. cerevisiae* or *Z. rouxii* proteins. Though *S. cerevisiae* Scc3 packed tightly as dimers in the crystals, those from *Z. rouxii* crystallized as monomers. Consistent with a monomeric conformation in solution, two differently tagged *Sc Scc3* proteins failed to co-precipitate when pure proteins were mixed in an equimolar ratio (Fig. 2-fig.sup.1B). Likewise, two differently tagged versions of the part of Scc1 that binds Scc3 (259–451) failed to co-precipitate in the presence of intact Scc3 protein (Fig. 2-fig.sup.1C). These observations imply that a single molecule of Scc3 binds Scc1, a finding that is inconsistent with the suggestion that through binding simultaneously two different Scc1 molecules, Scc3 mediates sister chromatid cohesion by holding two cohesin rings together [45]. Such a model is also inconsistent with the entrapment of sister DNAs within chemically circularized rings using bi-functional thiol-specific reagents to cross-link cysteine pairs inserted at the ring’s three interfaces [10].

## 4 Residues essential for cohesin’s release from chromatin

Cohesin is not stably associated with chromatin prior to DNA replication, with residence times varying from 2 to 15 min [4], [8], [9]. If association involves entrapment of DNAs within cohesin rings, then release must involve their subsequent escape through an exit gate situated at the Smc3–kleisin interface [4], a process that depends on Wapl [36]. Establishment of stable sister cohesion during S phase requires that the release process be inactivated, an event mediated by Eco1-dependent acetylation of Smc3 ATPase heads. Because abrogation of release is the sole essential function of Eco1, the acetyl transferase can be bypassed by mutations that reduce releasing activity. These include (spontaneous) point mutations in Wapl, Smc3, Pds5, and Scc3 [2], [30], [35]. Our structure reveals that the latter are confined to two small regions close to Scc3’s N- and C-termini. Thus, Scc3D189E and E202K are in juxtaposed α-helices at the N-terminus while R996S is situated in a loop closely juxtaposed to the penultimate α-helix containing R1043L (Fig. 3A). Gel filtration experiments showed that E202K had little or no effect on Scc3’s ability to bind Wapl (Fig. 3B and Fig. 3-fig.sup.1) or Scc1 (Fig. 3-fig.sup.1). Similar results were obtained with all four *eco1* suppressor mutations (data not shown). Indeed, Wapl’s recruitment to chromosomal cohesin complexes in vivo is unaffected by Scc3E202K [4]. How domains at the two ends of a 125 Å long protein both participate in cohesin’s release from chromatin is unclear but indicates that Scc3 forms a bridge between different parts of the cohesin complex.

## 5 Scc3 is required to maintain sister chromatid cohesion

Three pieces of evidence suggest that Scc3 is required for cohesin’s recruitment to chromatin. First, a GFP tagged version of Scc1 (Δ319–327) lacking sequences necessary for binding Scc3 (Fig. 2C) fails to form peri-centric barrels in G2/M cells [16]. Second, cohesin containing Smc1E1158Q, which initiates but cannot consummate the loading process, fails to accumulate at core centromeres in cells lacking Scc3 [16]. Third, cohesin lacking Scc3 cannot associate in a salt-resistant manner with DNA in an in vitro loading reaction [22]. To address whether Scc3 is also required after it has loaded onto chromosomes and formed sister chromatid cohesion, we compared the effect of inactivating Scc1 or Scc3 by shifting wild type or ts mutants to the restrictive temperature following prior arrest in G2/M at the permissive temperature. This revealed that sister chromatid cohesion detected by GFP-tagged Tet repressors bound to operators integrated at the *URA3* locus was lost in *scc3*–*1* as well as *scc1*–*73* cells (Fig. 4A). The implication is that Scc3 is required to maintain sister chromatid cohesion as well as establish it.

To view cohesin’s fate in live cells when Scc3 is inactivated, we created strains in which the endogenous *SCC1* or *SCC3* genes were tagged with the Auxin Inducible Degron (AID) [25]. *SCC3-AID* cells were incapable of proliferating in the presence of auxin (Fig. 4-fig.sup.1) and Western blotting showed that Scc1-PK3-AID and Scc3-PK3-AID were depleted within 30 min of adding auxin to cells arrested in G2/M phase by nocodazole (Fig. 4B and Fig. 4-fig.sup.2). Under these conditions, Scc1 depletion abolished co-localization of Scc3-GFP with centromeres in live cells while Scc3 depletion had a similar, albeit less complete, effect on Scc1-GFP (Fig 4C and Fig. 4-fig.sup.3). This suggests that Scc3 helps to maintain cohesin’s association with chromatin as well as load it onto chromosomes in the first place. These conclusions differ in certain respects from those using a different degron, which failed to inactivate Scc3 fully [18]. Though it triggered cohesin’s dissociation from centromeres, depletion of Scc3 or Scc1 in G2/M phase cells did not affect acetylation of Smc3K113 (Fig. 4B). Similarly, cleavage of a version of Scc1 containing three TEV sites by TEV protease induction in G2/M phase cells previously depleted of Scc3, also retained acetylation of Smc3K113 (Fig. 4-fig.sup.2). This was surprising because it contrasts with the effect of depleting Pds5, which causes rapid Hos1-dependent de-acetylation [3]. Whereas Pds5 is required to protect Smc3K113Ac from Hos1, Scc3 might be involved in promoting de-acetylation.

## 6 Unlike core ring subunits, Scc3 can turnover in post-replicative cells

Photo-bleaching experiments suggested that centromeric Scc3 turns over with kinetics similar to that of ring subunits such as Scc1 or Smc3 [4]. Acetylation of Smc3 prevents cohesin’s release in post-replicative cells and thereby helps maintain cohesion. Due to technical limitations, such experiments may fail to detect slow turnover of cohesin subunits within the complex. A more sensitive assay is to measure the ability of wild type protein synthesized during G2/M phase to suppress the loss of cohesion induced by shifting ts mutants to the restrictive temperature. As previously shown [14], induction of Scc1 from the *GAL1*–*10* promoter in cells arrested in metaphase cannot rescue loss of cohesion triggered by shifting *scc1–73* cells to the restrictive temperature (Fig. 4D). Likewise, expression of Smc1 failed to suppress loss of cohesion induced by *smc1–259* (Fig. 4D). Surprisingly, Scc3 expression was able to suppress loss of cohesion caused by *scc3–1* (A249E) (Fig. 4D and Fig. 4-fig.sup.4). The Smc1/Smc3, Smc/Scc1, and Smc3/Scc1 interfaces that create cohesin’s hetero-trimeric ring are all very stable, at least in the absence of specific mechanisms to disrupt them. As each of these subunits is bound to the complex by two strong interactions, subunit turnover may be impossible. Besides which, stable DNA entrapment may be incompatible with turnover of ring subunits. In contrast, only a single strong interaction holds Scc3 on the ring. Our findings suggest that this interaction has an off-rate in vivo that permits significant subunit turnover during extended periods of time in a manner that does not compromise sister chromatid cohesion.

## 7 The function of Scc3’s most highly conserved domain

To address the function of Scc3’s most highly conserved domain, we created a series of mutations at highly conserved positions (Fig. 5A and B). Surprisingly, most had little effect on cell proliferation (Fig. 5C). In contrast, D373R, R291E, R291A, and W389E single mutations all caused temperature sensitive growth (Fig. 5C and Fig. 5-fig.sup. 1), as did the double mutation R370E Y371E. W389 is clearly internal and its replacement may affect the packing of α-helices in this part of the protein but the other mutations affect residues closer to the surface. The lethality of these mutations implies that this highly conserved domain has an essential role; hence its name CES.

If the function of the CES is to interact with another macromolecule, either DNA or protein, then mutation of surface residues alone should also affect function. To our surprise, double or triple mutations substituting conserved residues on the surface of the protein did not cause lethality in mitotic cells. Thus, the simultaneous substitution of a pair of surface aromatic residues, namely Y371 and W408 by either alanine or glutamic acid was not lethal nor was the simultaneous substitution by either alanine or glutamic acid of three positively charged residues, namely K372, K404, and R449. Clearly, more extensive mutagenesis of surface residues within this highly conserved domain will be required to tease out its function. Interestingly, diploids homozygous for K372A K404A R449A and K372E K404E R449E triple mutations were inefficient in producing four spored asci. Moreover, the viability of spores from such asci was considerably lower than wild type (Fig. 5-fig.sup.2). A similar but more pronounced phenotype has been observed when analyzing diploids homozygous for Y371A W408A and Y371E W408E double mutants (data not shown). This suggests that the CES does indeed compromise Scc3 function, an effect that so far is more apparent during meiosis than mitosis.

It has been suggested on the basis of peptide arrays and co-immunoprecipitation studies that Scc3’s CES is concerned with binding cohesin’s loading complex Scc2/4 [22]. To test this hypothesis, we checked whether the CES is equally conserved in organisms that do not encode Scc2. We have previously reported the absence of an Scc2 ortholog in *Encephalitozoon* *cuniculi* [24] and have now confirmed their absence in a variety of other microsporidians (data not shown). Being intra-cellular fungal parasites, microsporidians have highly reduced genomes and have lost many supposedly essential genes. Interestingly, we also failed to identify Scc2 in the genomes of the red algae *Cyanidioschyzon* *merolae* and *Galdieria* *sulphuraria*, which are thought to have survived an evolutionary bottleneck during which they lost flagella and centrioles. Unlike microsporidians, red algae have retained both Pds5 and Eco1. Scc2 is a very large α-helical repeat protein with conservation throughout much of its length and its orthologs are normally easy to identify, even in trypanosomes. Its absence from microsporidians and red algae is therefore highly significant. Crucially, Scc3 proteins are present in both of these groups, underlying their fundamental role in cohesin biology. Moreover, they have retained conserved CES domains (Fig. 5A). It is therefore unlikely that the main function of Scc3’s CES is to interact with Scc2. Despite the presence of canonical CESs, Scc3 proteins in microsporidians are missing several of the helical repeats, including those that make up Scc3’s nose (Fig. 5D, Fig. 5-fig.sup.3 and Fig. 1A). Despite its absence in microsporidians, Scc3’s nose has an important function as its deletion (Δ306–359) causes lethality in yeast (Fig. 5E).

Scc3 has multiple roles in the biology of cohesin, in its loading, release, and maintenance on chromosomes. Unlike all other factors that regulate the behavior of cohesin’s Smc1/Smc3/Scc1 heterotrimeric ring, Scc3 is present in all eukaryotes. One of its mammalian orthologs, SA1, has been implicated in telomere replication and gene regulation [28], [29] while another, SA2, is particularly important for sister chromatid cohesion [33]. A third, STAG3, is crucial during meiosis [42]. Recent work suggests that mutations in SA2 are found in human tumours [32], [43]. The structure of Scc3 reported here will be invaluable for elucidating the molecular functions of this crucial cohesin subunit.

## 8 Materials and methods

### 8.1 Cloning, expression and purification for crystallography

Well-behaving domains of *S. cerevisiae* Scc3 (Uniprot SCC3_YEAST) were found using a trypsin digestion assay and mass spectrometry in combination with Edman N-terminal protein sequencing. Fragment *Sc* Scc3–9 was amplified by PCR and cloned into expression vector pHis17 (Bruno Miroux, personal communication), generating *Sc* Scc3 M-674-1072-H_6_ with no other additional residues present. C41(DE3) *E. coli* cells (Lucigen) were transformed and induced at OD_600_ 0.8 with 1 mM IPTG and grown for another 4–6 h at 25 °C. Cleared cell lysate in TAP buffer (50 mM Tris/HCl, 250 mM NaCl, 5 mM TCEP, pH 7.5) was pumped over nickel resin (GE Healthcare HisTrap) and eluted with 300 mM imidazole in TAP buffer. The eluate was diluted 10-fold with TAP and applied to Q resin (GE Healthcare HiTrap) and eluted with a gradient of 0–1 M NaCl in TAP buffer minus NaCl. After concentration in Centriprep centrifugal concentrators (Milipore, 10 kDa MWCO) the peak was applied to a size exclusion column (GE Healthcare Sephacryl S200) in TAP buffer. The final peak was concentrated as before to around 30 mg/ml and flash frozen in small aliquots. The identity of the protein was verified by electrospray masspec: 47,510 Da (calculated 47,507 Da). 120 l *E. coli* culture produced 62 mg of pure protein. Seleno methionine *Sc* Scc3–9 was produced using published procedures for feedback inhibition [39], [40] and purification followed the protocol for the native protein. Scc3 from *Z.* *rouxii* (NCBI XP_002497125.1) was optimized for expression and crystallization by trimming both N- and C-termini to remove unstructured regions. The final construct was synthesized and codon optimized (Genscript, Hong Kong) and cloned into expression vector pET21a generating (*Zr* Scc3 M-88-1072-H_6_). BL21AI cells (Lifetechnologies) were transformed and induced at OD_600_ 0.8 with 0.2% arabinose, and further grown over night at 15 °C. Cleared cell lysate in TAP buffer was pumped over nickel resin (GE Healthcare HisTrap) and eluted with 300 mM imidazole in TAP. The peak was concentrated using Centriprep concentrators (Millipore, 10 kDa MWCO) and applied to a size exclusion column in TAP buffer (GE Healthcare Sephacryl S300). The resulting peak was concentrated as before to 15 mg/ml and flash frozen in liquid nitrogen in small aliquots. The identity of the protein was verified by electrospray mass spectrometry: 109,632 Da (calculated minus M1: 109,614 Da). 12 l *E. coli* culture produced 6 mg of pure protein. Selenomethionine *Zr* Scc3 was produced using published procedures for feedback inhibition [39], [40] and purification followed the protocol for the native protein as described with the following changes: C41(DE3) cells were used, IPTG was used as inducer and extra care needed to be taken in order to avoid proteolysis of the protein during cell lysis and purification.

### 8.2 Structure determination

Crystallization conditions were found using our in house nanolitre crystallization facility [34]. For *Sc* Scc3–9, crystals were grown by vapor diffusion, equilibrating drops of 100 nl reservoir plus 100 nl protein solution (30 mg/ml) against reservoir containing 100 mM cacodylate/EtOOH pH 6.5, 7.5% (w/v) PEG 20,000, 15% (w/v) PEG 550 MME, 400 mM KSCN and 2.5% (w/v) jeffamine M-600 (adjusted to pH 7). Crystals were cryo-protected by adding reservoir solution supplemented with 15% glycerol, before flash freezing in liquid nitrogen. Datasets were collected at ESRF (Grenoble, France) on beamline id23eh1 and indexed and integrated with iMOSFLM [1]. Data reduction was performed with SCALA [41]. Phasing was performed with a SeMet SAD experiment using isomorphous crystals obtained under identical crystallization conditions. Selenium sites were located using ShelxCDE [31] and phases were calculated with PHASER in SAD mode [21]. A model was automatically built with BUCCANEER [7], manually adjusted using MAIN [38] and refined with REFMAC at 2.1 Å resolution [23]. For *Zr* Scc3, reservoir solution used contained 100 mM Morpheus Buffer 1. pH 6.5, 0.15 × Morpheus carboxylic acid mix (Molecular Dimensions), 15% (w/v) PEG 4000 and 30% (v/v) glycerol [11]. *Zr* Scc3 crystals were flash frozen in liquid nitrogen without adding further cryoprotectant. Datasets were collected at Diamond Light Source (Harwell, UK) on beamlines I04-1 and I04 and indexed and integrated with XDS [17]. All further structure determination procedures were essentially performed as for *Sc* Scc3–9. The structure and structure factors have been deposited in the Protein Data Bank (PDB) with accession codes 4UVJ and 4UVK for *Sc* Scc3–9 and *Zr* Scc3 respectively and statistics of the data and models are summarized in Table 1.

### 8.3 Protein purification for pull downs

*E. coli* BL21(DE3) RIPL cells (Stratagene) were transformed with pET vectors containing the *S. cerevisiae* gene of interest and induced at OD_600_ 0.6 with 1 mM IPTG at 23 °C over night. The cells were pelleted and re-suspended with TAP buffer (50 mM Tris–HCl, 250 mM NaCl, 1 mM β-mercaptoethanol, pH 7.5) adding EDTA-free protease inhibitor cocktail tablets (Roche). After re-suspension, and one round of cell lysis at 18 KPsi (Constant Systems), 1 min sonication at 80% AMPL (Sonics Vibra-Cell) were performed. Cell lysate was pre-cleared by centrifugation at 4000 rpm for 15 min, and supernatant was cleared by centrifugation at 15,000 rpm for 1 h at 4 °C (Beckman Coulter, rotor JLA-16.250). All purified proteins are His_6_ tagged and supernatants were incubated with Talon Superflow beads (Clontech) for 3 h at 4 °C. Beads were washed 3 times with TAP buffer containing 10 mM imidazole. Proteins were eluted in TAP buffer with 500 mM imidazole and loaded onto a Superdex 200 16/600 chromatography column (GE Healthcare) equilibrated with TAP buffer. Peak fractions were collected and concentrated using Vivaspin columns (Sartorius Stedim Biotech). To obtain the Smc ATPase heads with the Scc1 (310–566) fragment complex, pET28a-Smchd-Strep and pET21d-His_6_-Scc1(310–566)-Strep were co-transformed, co-expressed and purified using Talon Superflow beads (Clontech) as described above.

### 8.4 Pull downs

5 μM of each purified protein was incubated in a final volume of 100 μl TAP buffer. 10% input sample (i) prior incubation was removed, adjusted to 45 μl with Laemmli sample buffer and boiled. The rest of the sample was incubated with 50 μl of 50% (v/v) of pre-washed resin in TAP buffer (Strep-tactin, IBA; anti-Flag M2 affinity gel, Sigma) for 2 h at 4 °C. Following the incubation and a short spin (1 min, 2000 rpm), 10% of supernatant was removed and adjusted to 45 μl with Laemmli sample buffer and boiled, generating the flow-through sample (ft). The pelleted resin was washed 3 times with 1 ml TAP buffer and finally re-suspended in 45 μl Laemmli sample buffer and boiled, making the bound sample (b). 10 μl of each collected samples were analyzed by 12% SDS–PAGE and Coomassie staining, or Western blotting.

### 8.5 Gel filtration binding assays

For in vitro binding assays, 15 μM of each pure protein were incubated at 4 °C for 2–3 h, at a final volume of 150 μl in TAP buffer and then loaded onto a Superdex 200 10/300 column. Protein fractions were collected and analyzed by 12% SDS–PAGE and Coomassie staining.

### 8.6 Microscopy

GFP dot assays were performed as described as previously described [12]. All strains used for live-cell imaging were diploid cells. They were placed on 2.5% agarose pads made of synthetic complete medium plus glucose. Live cell imaging was performed under a spinning disk confocal system (Perkin Elmer UltraVIEW) with an EMCCD camera (Hamamatsu) mounted on an Olympus IX8 microscope with an Olympus 100 × 1.35 N.A. objective (Table 2).

**Table 2:** List of strains.

*All yeast strains are derivatives of W303*	
K699	*MATa*, *ade2–1*, *trp1–1*, *can1–100*, *leu2–3*,*112*,*his3–11*,*15*, *ura3*, *GAL*, *psi+*
K7606	*MATa*, *Scc3::HA3::HIS3*
K8266	*MATa*, *Scc1-HA3::HIS3*
K11990	*MATa*, *Scc1-PK6::KanMX*
K12568	*MATalpha*, *SMC1-myc18::TRP1*
K15024	*MATa*, *tetR-GFP::LEU2*, *tetOs::URA3*, *TRP1::Met3-Cdc20*
K15031	*MATa*, *scc1–73*, *tetR-GFP::LEU2*, *tetOs::URA3*, *TRP1::Met3-Cdc20*
K15071	*MATa*, *scc3–1*, *tetR-GFP::LEU2*, *tetOs::URA3*, *TRP1::Met3-Cdc20*
K16524	*MATalpha*, *GAL1-SCC1::TRP1 at SCC1*, *leu::Pssc1-SCC1(deletion328–348)-HA3::LEU2*
K16525	*MATalpha*, *GAL1-SCC1::TRP1 at SCC1*, *leu::Pssc1-SCC1(deletion349–369)-HA3::LEU2*
K16526	*MATalpha*, *GAL1-SCC1::TRP1 at SCC1*, *leu::Pssc1-SCC1(deletion370–393)-HA3::LEU2*
K16677	*MATa*, *pGAL1–10::TRP*, *tetR-GFP::LEU2*, *tetOs::URA3*, *TRP1::Met3-Cdc20*
K16678	*MATa*, *scc3–1*, *pGAL1–10-Scc3-HA3::TRP*, *tetR-GFP::LEU2*, *tetOs::URA3*, *TRP1::Met3-Cdc20*
K16679	*MATa*, *smc1–259*, *pGal1–10-Smc1-myc18::LEU2*, *tetR-GFP::LEU2*, *tetOs::URA3*, *TRP1::Met3-Cdc20*
K16680	*MATa*, *scc1–73*, *pGAL1–10-Scc1-HA3::LEU2*, *tetR-GFP::LEU2*, *tetOs::URA3*, *TRP1::Met3-Cdc20*
K17128	*MATalpha*, *GAL1-SCC1::TRP1 at SCC1*, *leu::Pscc1-SCC1(del319–327)-HA3::LEU2*
K17696	*MATa*, *ura::ADH1 promoter-OsTIR1–9myc::URA3*
K18154	*MATa ura::ADH1 promoter-OsTIR1–9myc::URA3*, *Scc1-Pk3-aid::KanMX4*
K20316	*MATa*, *ura::ADH1 promoter-OsTIR1–9myc::URA3*, *Scc3-PK3-aid::KanMX4*
K20783	*MATa*, *ura::ADH1 promoter-OsTIR1–9myc::URA3*, *Scc3-PK3-aid::KanMX4*, *Scc1-HA3::HIS3*
K20785	*MATa*, *Scc3-PK3-aid::KanMX4*, *Scc1-HA3::HIS3*
K20787	*MATa*, *ura::ADH1 promoter-OsTIR1–9myc::URA3*, *Scc1-Pk3-aid::KanMX4*, *Scc3::HA3::HIS*
K20789	*MATa*, *Scc1-Pk3-aid::KanMX4*, *Scc3::HA3::HIS*
K20791	*MATa*, *Scc1-Pk3-aid::KanMX4*
K20795	*MATa*, *Scc3-PK3-aid::KanMX4*
K20854	*MATa/alpha*, *ura::ADH1 promoter-OsTIR1–9myc::URA3*, *SCC1EGFP::HIS3*, *Scc3-Pk3-aid::KanMX4*, *Mtw1-RFP:KanMX*, *ADE2*
K21329	*MATa*, *scc3::NatMX4*, *leu::Scc3(R370E)-HA3::LEU2*, *Scc1-PK6::TRP1*, *Mtw1-RFP:KanMX*, *ADE2*
K21443	*MATa*, *scc3::NatMX4*, *leu::Scc3(W389E)-HA3::LEU2*, *Scc1-PK6::TRP1*, *Mtw1-RFP:KanMX*, *ADE2*
K21713	*MATa*, *scc3::NatMX4*, *leu::Scc3(R291E)-HA3::LEU2*, *Scc1-PK6::TRP1*, *Mtw1-RFP:KanMX*, *ADE2*
K21719	*MATa*, *scc3::NatMX4*, *leu::Scc3(D373R)-HA3::LEU2*, *Scc1-PK6::TRP1*, *Mtw1-RFP:KanMX*, *ADE2*
K21777	*MATa/alpha scc3::NatMX4/WT*, *leu/leu::LEU2 (YIplac128 integrated)*, *Scc1-PK6::TRP1*, *Mtw1-RFP:KanMX ADE2*
K21815	*MATa/alpha scc3::NatMX4/WT*, *Scc1-PK6::TRP1*, *leu::Scc3-HA3::LEU2*, *Mtw1-RFP:KanMX*, *ADE2*
K21817	*MATa*, *scc3::NatMX4*, *leu::Scc3-HA3::LEU2*, *Scc1-PK6::TRP1*, *Mtw1-RFP:KanMX*, *ADE2*
K23026	*MATa*, *scc3::NatMX4*, *leu::Scc3(R370E*, *Y371E)-HA3::LEU2*, *Scc1-PK6::TRP1*, *Mtw1-RFP:KanMX*, *ADE2*
K23048	*MATa scc3–1 (A249E)*
K23050	*MATa*, *ura::ADH1 promoter-OsTIR1–9myc::URA3*, *Scc3-PK3-aid::KanMX4*, *Scc1(TEV3)220-HA6::HIS3*, *leu2::His3p-Gal1/His3p-Gal2/Gal1p-Gal4::Leu2*, *YEplac112 GAL-NLS2-TEV-NLS*
K23181	*MATa/alpha*, *ura::ADH1 promoter-OsTIR1–9myc::URA3*, *Scc1-Pk3-AID::KanMX4*, *SCC3EGFP::HIS*, *Mtw1-RFP:KanMX*, *ADE2*
K23235	*MATa*, *scc3::NatMX4*, *leu::Scc3(R291A)-HA3::LEU2*, *Scc1-PK6::TRP1*, *Mtw1-RFP:KanMX*, *ADE2*
K23289	*MATa*, *scc3::NatMX4*, *leu::Scc3(Y371E)-HA3::LEU2*, *Scc1-PK6::TRP1*, *Mtw1-RFP:KanMX*, *ADE2*
K23304	*MATa*, *scc3::NatMX4*, *leu::Scc3 (120–1060)HA3::LEU2*, *Scc1-PK6::TRP1*, *Mtw1*, *RFP:KanMX*, *ADE2*
K23306	*MATa/alpha leu::Scc3(306–359delta)HA3::LEU2*, *Scc1-PK6::TRP1*, *Mtw1-RFP:KanMX*, *ADE2*
K21815	*MATa/alpha*, *scc3::NatMX4/WT*, *Scc1-PK6::TRP1*, *leu::Scc3-HA3::LEU2*, *Mtw1-RFP:KanMX*, *ADE2*
K21817	*MATa*, *scc3::NatMX4*, *Scc1-PK6::TRP1*, *leu::Scc3-HA3::LEU2*, *Mtw1-RFP:KanMX*, *ADE2*
K21818	*MATalpha*, *scc3::NatMX4*, *Scc1-PK6::TRP1*, *leu::Scc3-HA3::LEU2*, *Mtw1-RFP:KanMX*, *ADE2*
K23276	*MATa/alpha*, *scc3::NatMX4/WT*, *leu::Scc3(K372A*, *K404A*, *R449A)-HA3::LEU2*, *Scc1-PK6::TRP1*, *Mtw1-RFP:KanMX*, *ADE2*
K23277	*MATa*, *scc3::NatMX4/WT*, *leu::Scc3(K372A*, *K404A*, *R449A)-HA3::LEU2*, *Scc1-PK6::TRP1*, *Mtw1-RFP:KanMX*, *ADE2*
K23278	*MATalpha*, *scc3::NatMX4*, *leu::Scc3(K372A*, *K404A*, *R449A)-HA3::LEU2*, *Scc1-PK6::TRP1*, *Mtw1-RFP:KanMX*, *ADE2*
K23279	*MATa/alpha scc3::NatMX4/WT*, *leu::Scc3(K372E*, *K404E*, *R449E)-HA3::LEU2*, *Scc1-PK6::TRP1*, *Mtw1-RFP:KanMX*, *ADE2*
K23280	*MATa*, *scc3::NatMX4*, *leu::Scc3(K372E*, *K404E*, *R449E)-HA3::LEU2*, *Scc1-PK6::TRP1*, *Mtw1-RFP:KanMX ADE2*
K23281	*MATalpha*, *scc3::NatMX4*, *leu::Scc3(K372E*, *K404E*, *R449E)-HA3::LEU2*, *Scc1-PK6::TRP1*, *Mtw1-RFP:KanMX*, *ADE2*
K23382	*MATa/alpha*, *SCC1EGFP::HIS3*, *Scc3-PK3-aid::KanMX4*, *ADE2*
K23385	*MATa/alpha*, *SCC1-Pk3-aid::KanMX4*, *SCC3EGFP::HIS3*, *ADE2*

### 8.7 Yeast culture and cohesion assay

Unless otherwise stated, cells were grown in YEPD medium. For G1 phase arrest, cells were incubated in 5 μg/ml α-factor peptide for 90 min starting at OD_600_ = 0.2. Strains with the CDC20 gene under control of the MET3 promoter were grown in minimal medium lacking methionine and arrested in mitosis in YEP supplemented with 2 mM methionine. For G2/M arrest, cells were incubated with 10 μg/ml nocodazole for 2 h.

### 8.8 Cell viability of *SCC1* deletions

Ectopic *scc1* mutants under its native promoter were incorporated at the *leu2* locus in cells whose endogenous wild type gene *SCC1* is under the control of Gal1–10 promoter. Viability analysis was measured by streaking transformed cells on YEP glucose plates, switching off wild type SCC1 expression.

### 8.9 Cell viability of Scc3 mutants/deletions

Mutant versions of *scc3* (under its native promoter) were incorporated at the *leu2* locus in heterozygous *SCC3/scc3Δ* diploids cells. Diploids were sporulated and tetrads dissected at 23 °C on YPD plates. The genotype of the resulting haploids was determined by replica plating, and viable cells with only the ectopic copy were additionally tested for temperature sensitivity streaking cells on YEP glucose plates. All mutations were confirmed by DNA sequencing.

## References

[1] Battye T.G., Kontogiannis L., Johnson O., Powell H.R., Leslie A.G. (2011). IMOSFLM: a new graphical interface for diffraction-image processing with MOSFLM. Acta Crystallogr. D.

[2] Ben-Shahar T.R., Heeger S., Lehane C., East P., Flynn H., Skehel M., Uhlmann F. (2008). Eco1-dependent cohesin acetylation during establishment of sister chromatid cohesion. Science.

[3] Chan K.L., Gligoris T., Upcher W., Kato Y., Shirahige K., Nasmyth K., Beckouet F. (2013). Pds5 promotes and protects cohesin acetylation. Proc. Natl. Acad. Sci. USA.

[4] Chan K.L., Roig M.B., Hu B., Beckouet F., Metson J., Nasmyth K. (2012). Cohesin’s DNA exit gate is distinct from its entrance gate and is regulated by acetylation. Cell.

[5] Chatterjee A., Zakian S., Hu X.W., Singleton M.R. (2013). Structural insights into the regulation of cohesion establishment by Wpl1. EMBO J..

[6] Ciosk R., Shirayama M., Shevchenko A., Tanaka T., Toth A., Nasmyth K. (2000). Cohesin’s binding to chromosomes depends on a separate complex consisting of Scc2 and Scc4 proteins. Mol. Cell.

[7] Cowtan K. (2006). The Buccaneer software for automated model building. 1. Tracing protein chains. Acta Crystallogr. D.

[8] Eichinger C.S., Kurze A., Oliveira R.A., Nasmyth K. (2013). Disengaging the Smc3/kleisin interface releases cohesin from *Drosophila* chromosomes during interphase and mitosis. EMBO J..

[9] Gerlich D., Koch B., Dupeux F., Peters J.M., Ellenberg J. (2006). Live-cell imaging reveals a stable cohesin–chromatin interaction after but not before DNA replication. Curr. Biol..

[10] T. Gligoris, J. Scheinost, P. Uluacak, N. Petela, K. Chan, F. Bürmann, F. Beckouet, S. Gruber, K. Nasmyth, J. Löwe, Closing the cohesin ring: structure and function of its Smc3–Kleisin interface, Science (2014) (Submitted for publication). 10.1126/science.1256917 PMC4300515 25414305

[11] Gorrec F. (2009). The MORPHEUS protein crystallization screen. J. Appl. Crystallogr..

[12] Gruber S., Arumugam P., Katou Y., Kuglitsch D., Helmhart W., Shirahige K., Nasmyth K. (2006). Evidence that loading of cohesin onto chromosomes involves opening of its SMC hinge. Cell.

[13] Haering C.H., Farcas A., Arumugam P., Metson J., Nasmyth K. (2008). The cohesin ring concatenates sister DNAs. Nature.

[14] Haering C.H., Schoffnegger D., Nishino T., Helmhart W., Nasmyth K., Lowe J. (2004). Structure and stability of cohesin’s Smc1–kleisin interaction. Mol. Cell.

[15] Hauf S., Roitinger E., Koch B., Dittrich C.M., Mechtler K., Peters J.M. (2005). Dissociation of cohesin from chromosome arms and loss of arm cohesion during early mitosis depends on phosphorylation of SA2. PLoS Biol..

[16] Hu B., Itoh T., Mishra A., Katoh Y., Chan K.L., Upcher W., Godlee C., Roig M.B., Shirahige K., Nasmyth K. (2011). ATP hydrolysis is required for relocating cohesin from sites occupied by its Scc2/4 loading complex. Curr. Biol..

[17] Kabsch W. (2010). XDS. Acta Crystallogr. D.

[18] Kulemzina I., Schumacher M.R., Verma V., Reiter J., Metzler J., Failla A.V., Lanz C., Sreedharan V.T., Ratsch G., Ivanov D. (2012). Cohesin rings devoid of Scc3 and Pds5 maintain their stable association with the DNA. PLoS Genet..

[19] Kurze A., Michie K.A., Dixon S.E., Mishra A., Itoh T., Khalid S., Strmecki L., Shirahige K., Haering C.H., Lowe J. (2011). A positively charged channel within the Smc1/Smc3 hinge required for sister chromatid cohesion. EMBO J..

[20] Losada A., Yokochi T., Kobayashi R., Hirano T. (2000). Identification and characterization of SA/Scc3p subunits in the *Xenopus* and human cohesin complexes. J. Cell Biol..

[21] McCoy A.J., Grosse-Kunstleve R.W., Adams P.D., Winn M.D., Storoni L.C., Read R.J. (2007). Phaser crystallographic software. J. Appl. Crystallogr..

[22] Murayama Y., Uhlmann F. (2014). Biochemical reconstitution of topological DNA binding by the cohesin ring. Nature.

[23] Murshudov G.N., Vagin A.A., Dodson E.J. (1997). Refinement of macromolecular structures by the maximum-likelihood method. Acta Crystallogr. D.

[24] Nasmyth K., Schleiffer A. (2004). From a single double helix to paired double helices and back. Philos. Trans. R. Soc. Lond. B.

[25] Nishimura K., Fukagawa T., Takisawa H., Kakimoto T., Kanemaki M. (2009). An auxin-based degron system for the rapid depletion of proteins in nonplant cells. Nat. Methods.

[26] Ouyang Z., Zheng G., Song J., Borek D.M., Otwinowski Z., Brautigam C.A., Tomchick D.R., Rankin S., Yu H. (2013). Structure of the human cohesin inhibitor Wapl. Proc. Natl. Acad. Sci. USA.

[27] Peters J.M., Tedeschi A., Schmitz J. (2008). The cohesin complex and its roles in chromosome biology. Genes Dev..

[28] Remeseiro S., Cuadrado A., Carretero M., Martinez P., Drosopoulos W.C., Canamero M., Schildkraut C.L., Blasco M.A., Losada A. (2012). Cohesin-SA1 deficiency drives aneuploidy and tumourigenesis in mice due to impaired replication of telomeres. EMBO J..

[29] Remeseiro S., Cuadrado A., Gomez-Lopez G., Pisano D.G., Losada A. (2012). A unique role of cohesin-SA1 in gene regulation and development. EMBO J..

[30] Rowland B.D., Roig M.B., Nishino T., Kurze A., Uluocak P., Mishra A., Beckouet F., Underwood P., Metson J., Imre R. (2009). Building sister chromatid cohesion: smc3 acetylation counteracts an antiestablishment activity. Mol. Cell.

[31] Sheldrick G.M. (2008). A short history of SHELX. Acta Crystallogr. A.

[32] Solomon D.A., Kim J.S., Bondaruk J., Shariat S.F., Wang Z.F., Elkahloun A.G., Ozawa T., Gerard J., Zhuang D., Zhang S. (2013). Frequent truncating mutations of STAG2 in bladder cancer. Nat. Genet..

[33] Solomon D.A., Kim T., Diaz-Martinez L.A., Fair J., Elkahloun A.G., Harris B.T., Toretsky J.A., Rosenberg S.A., Shukla N., Ladanyi M. (2011). Mutational inactivation of STAG2 causes aneuploidy in human cancer. Science.

[34] Stock D., Perisic O., Lowe J. (2005). Robotic nanolitre protein crystallisation at the MRC laboratory of molecular biology. Prog. Biophys. Mol. Biol..

[35] Sutani T., Kawaguchi T., Kanno R., Itoh T., Shirahige K. (2009). Budding yeast Wpl1(Rad61)–Pds5 complex counteracts sister chromatid cohesion-establishing reaction. Curr. Biol..

[36] Tedeschi A., Wutz G., Huet S., Jaritz M., Wuensche A., Schirghuber E., Davidson I.F., Tang W., Cisneros D.A., Bhaskara V. (2013). Wapl is an essential regulator of chromatin structure and chromosome segregation. Nature.

[37] Toth A., Ciosk R., Uhlmann F., Galova M., Schleifer A., Nasmyth K. (1999). Yeast cohesin complex requires a conserved protein, Eco1p (Ctf7), to establish cohesion between sister chromatids during DNA replication. Genes Dev..

[38] Turk D. (2013). MAIN software for density averaging, model building, structure refinement and validation. Acta Crystallogr. D.

[39] van den Ent F., Lockhart A., Kendrick-Jones J., Lowe J. (1999). Crystal structure of the N-terminal domain of MukB: a protein involved in chromosome partitioning. Structure.

[40] Van Duyne G.D., Standaert R.F., Karplus P.A., Schreiber S.L., Clardy J. (1993). Atomic structures of the human immunophilin FKBP-12 complexes with FK506 and rapamycin. J. Mol. Biol..

[41] Winn M.D., Ballard C.C., Cowtan K.D., Dodson E.J., Emsley P., Evans P.R., Keegan R.M., Krissinel E.B., Leslie A.G., McCoy A. (2011). Overview of the CCP4 suite and current developments. Acta Crystallogr. D.

[42] Winters T., McNicoll F., Jessberger R. (2014). Meiotic cohesin STAG3 is required for chromosome axis formation and sister chromatid cohesion. EMBO J..

[43] Yoshida K., Toki T., Okuno Y., Kanezaki R., Shiraishi Y., Sato-Otsubo A., Sanada M., Park M.J., Terui K., Suzuki H. (2013). The landscape of somatic mutations in down syndrome-related myeloid disorders. Nat. Genet..

[44] Zhang N., Jiang Y., Mao Q., Demeler B., Tao Y.J., Pati D. (2013). Characterization of the interaction between the cohesin subunits Rad21 and SA1/2. PLoS One.

[45] Zhang N., Kuznetsov S.G., Sharan S.K., Li K., Rao P.H., Pati D. (2008). A handcuff model for the cohesin complex. J. Cell Biol..

