# Supplementary material for: Structure and function of cohesin’s Scc3/SA regulatory subunit
Source: FEBS Lett. 2014 Oct 16;588(20):3692–702. doi: 10.1016/j.febslet.2014.08.015 (PMC4175184; doi:10.1016/j.febslet.2014.08.015)
Supplement: Supplementary data 1 [file mmc1.pdf]

**Supplementary Figures:**

# Structure and function of cohesin's Scc3/SA regulatory subunit

Maurici B Roig[a, 1], Jan Löwe[b, 1, *], Kok-Lung Chan[a] , Frédéric Beckövet[a], Jean Metson[a], and Kim Nasmyth[a, *]

[a] Department of Biochemistry, University of Oxford, Oxford OX1 3QU, United Kingdom

[b] MRC Laboratory of Molecular Biology, Structural Studies Division, Francis Crick Avenue, Cambridge CB2 0QH, United Kingdom.

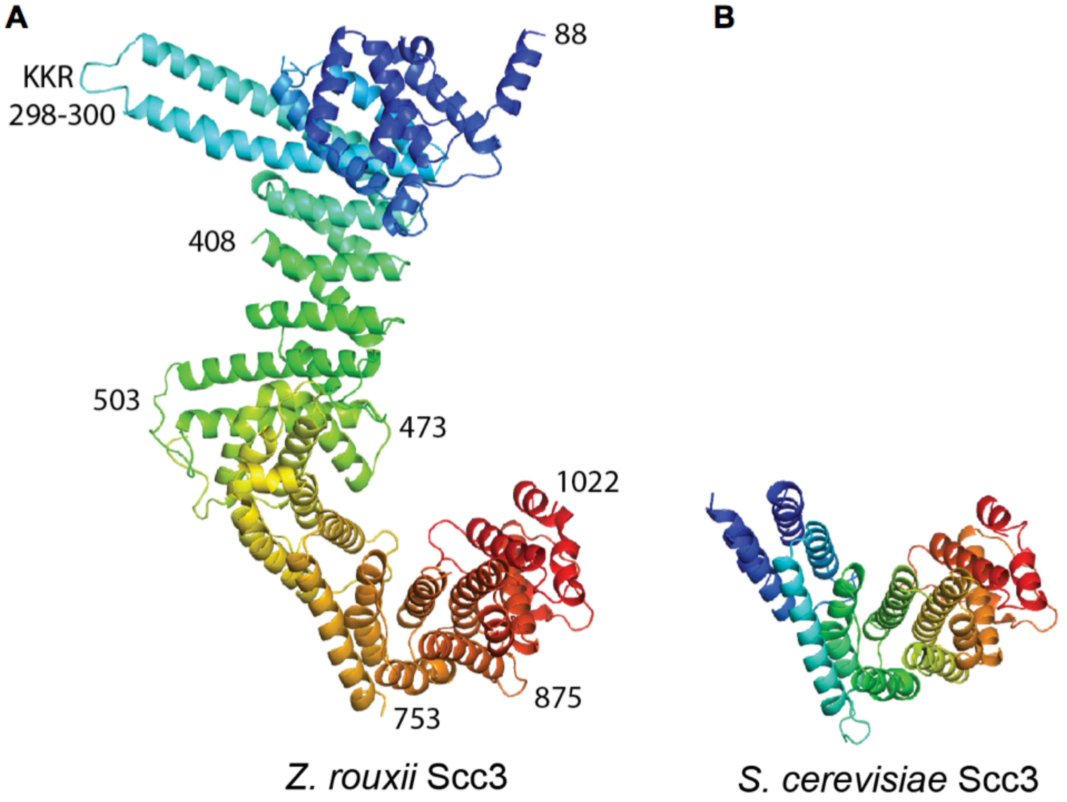


**Figure 1-figure supplement 1. Similarity of *Sc* Scc3 C-terminal fragment Scc3-9 and *Zr* Scc3.**

(A) *Zr* Scc3 structure, same as Fig. 1A, middle, colored blue to red from N- to C-terminus. (B) *Sc* Scc3 C-terminal fragment Scc3-9 showing basically the same structure as the equivalent region in (A), demonstrating that *Sc* and *Zr* Scc3 share the same fold. RMSD = 1.6 Å.

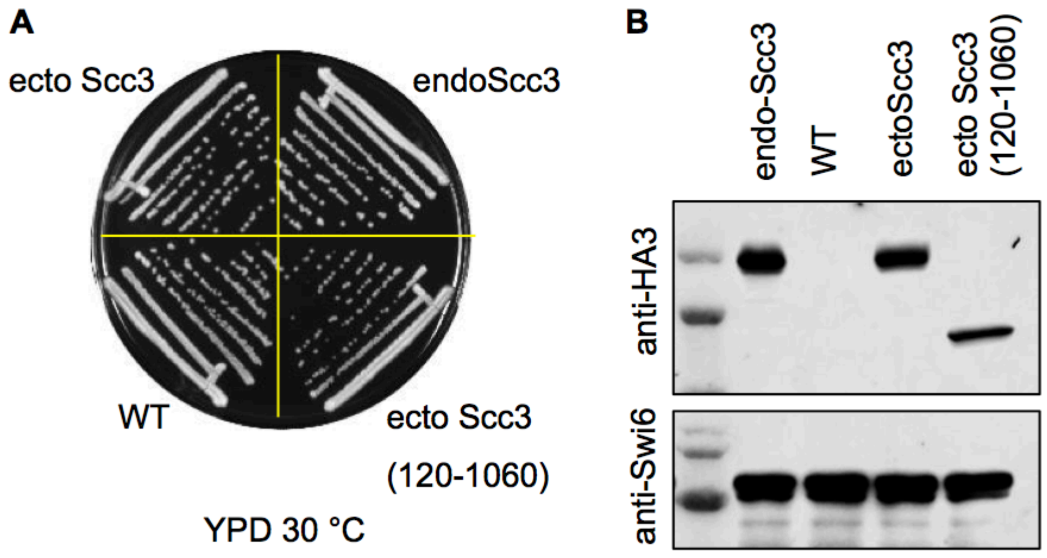


**Figure 1-figure supplement 2. S1. N- and C-terminal ends of *Sc* Scc3 are not essential.**

(A) HA3 tagged *SCC3* genes expressing either full length *Sc* Scc3 or an Scc3 fragment (120-1060) missing Scc3's N- and C-terminal extensions, which corresponds to the crystallized *Zr* Scc3 (88-1022), was integrated at the ectopic *leu2* locus (ecto Scc3) of a heterozygous *SCC3/scc3Δ* diploid. Tetrad dissection yielded strains lacking the endogenous *SCC3* gene (i.e. *scc3Δ*) kept alive by either the ectopic wild type *SCC3* (K21817) or truncated version (120-1060) (K23304). On a YPD plate at 30°C, they show comparable growth to cells expressing the endogenous HA3 tagged *SCC3* gene (endo Scc3) (K7606) or *SCC3* wild type non tagged cells (K11990). (B) Western blots showing expression of ectopic wild type or truncated Scc3 HA3 tagged protein. Swi6 was loading control.

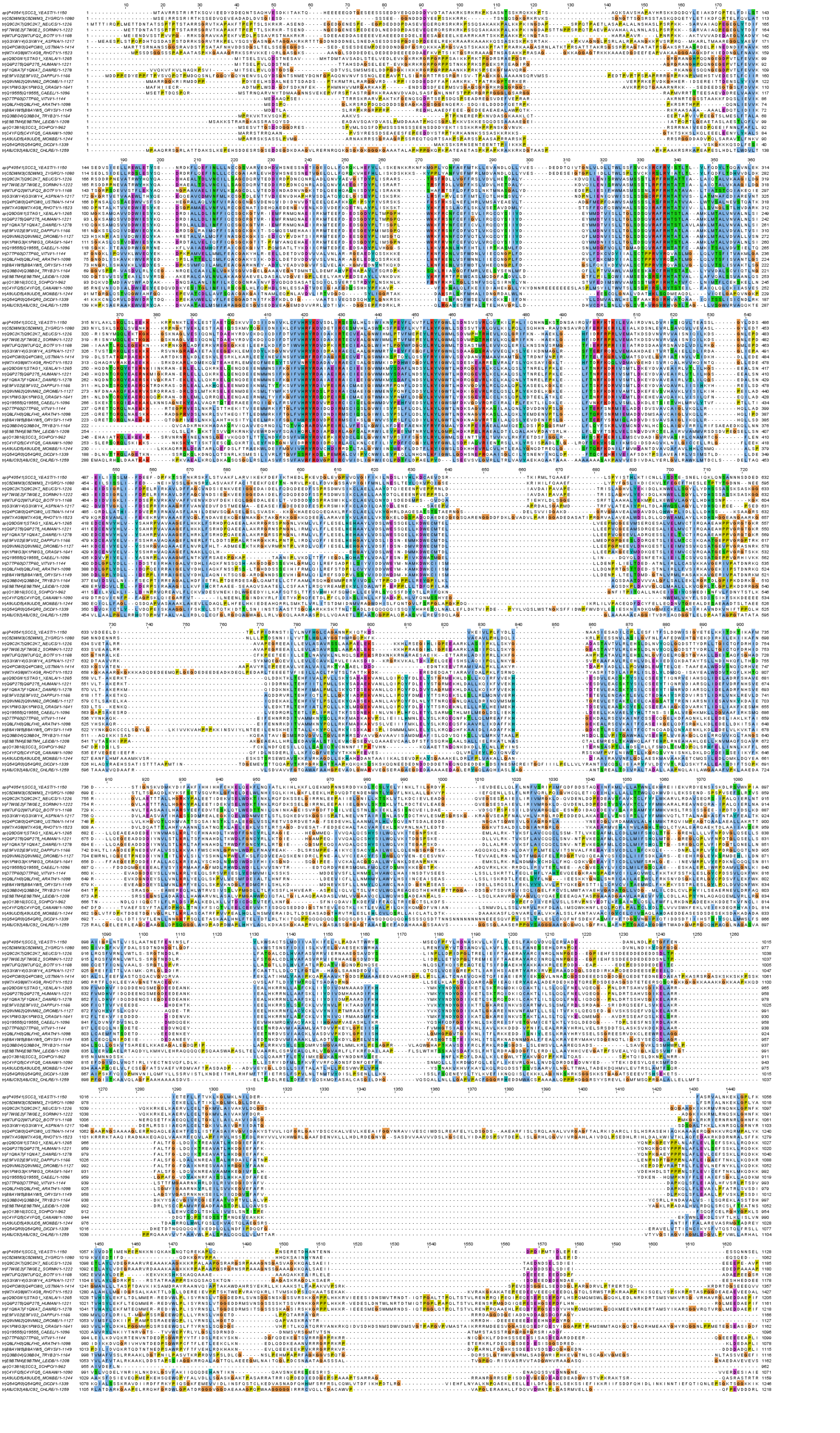

**Figure 1-figure supplement 3. Sequence alignment of eukaryotic Scc3 orthologs.**
A multiple sequence alignment (ClustalW) of evolutionary divergent eukaryotic organisms demonstrates conservation of the sequence contained in the crystallized fragment Zr Scc3 (88-1022). Note that the N-terminal half of the protein is more conserved than the C-terminal half, especially the conserved essential surface (CES) (Fig. 5).

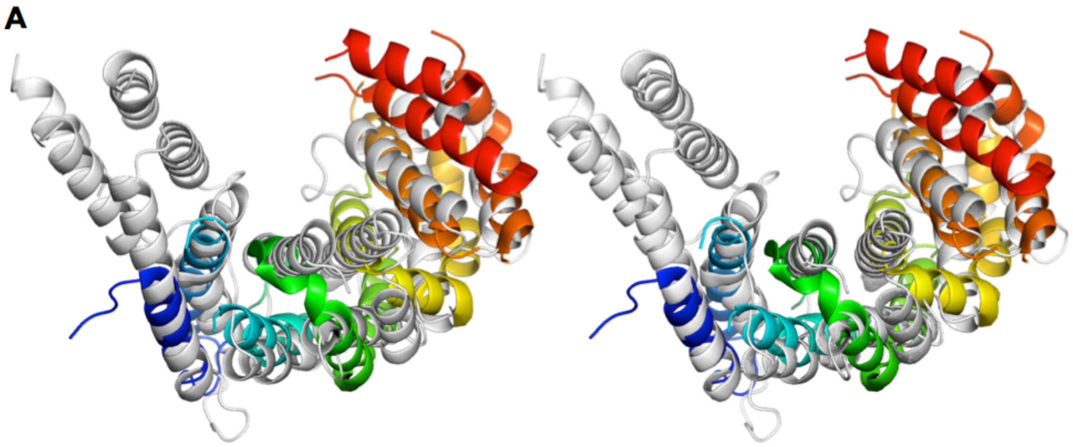


*Sc* Scc3-9 vs. PDB 4KF8 chain B; (RMSD ~ 4 Å); stereo

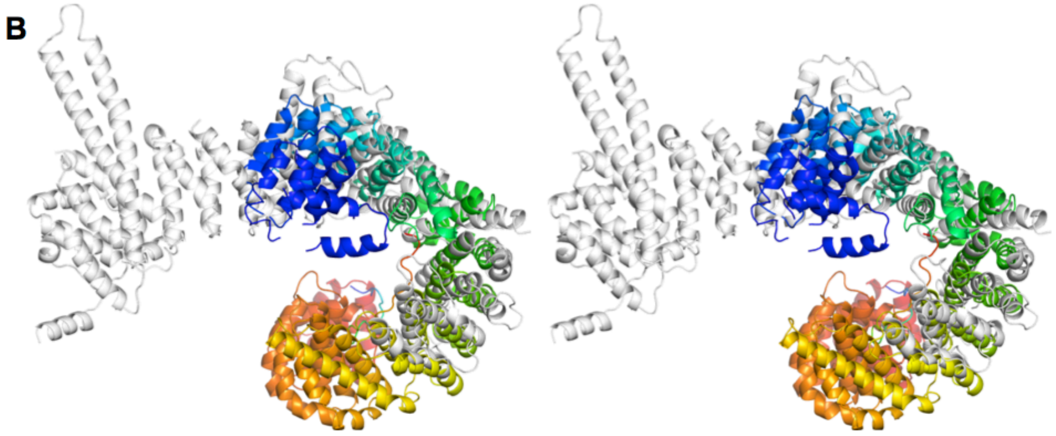


*Zr* Scc3 vs. PDB 2H4M (transportin); (RMSD ~ 4 Å); stereo

**Figure 1-figure supplement 4. Structural homologues of Scc3.**

DALI and PDBe Fold (SSM) searches of the Protein Dada Bank (PDB) revealed a long list of partial structural homologs of our Scc3 structures. As examples, (A) shows the superposition of *Sc* Scc9 and 4FK8 (nucleoporin Nup188) and (B) the superposition of Zr Scc3 and 2H4M (transportin/karyopherin). In both cases topology is conserved, but overall architecture is not, leading to high RMSD values.

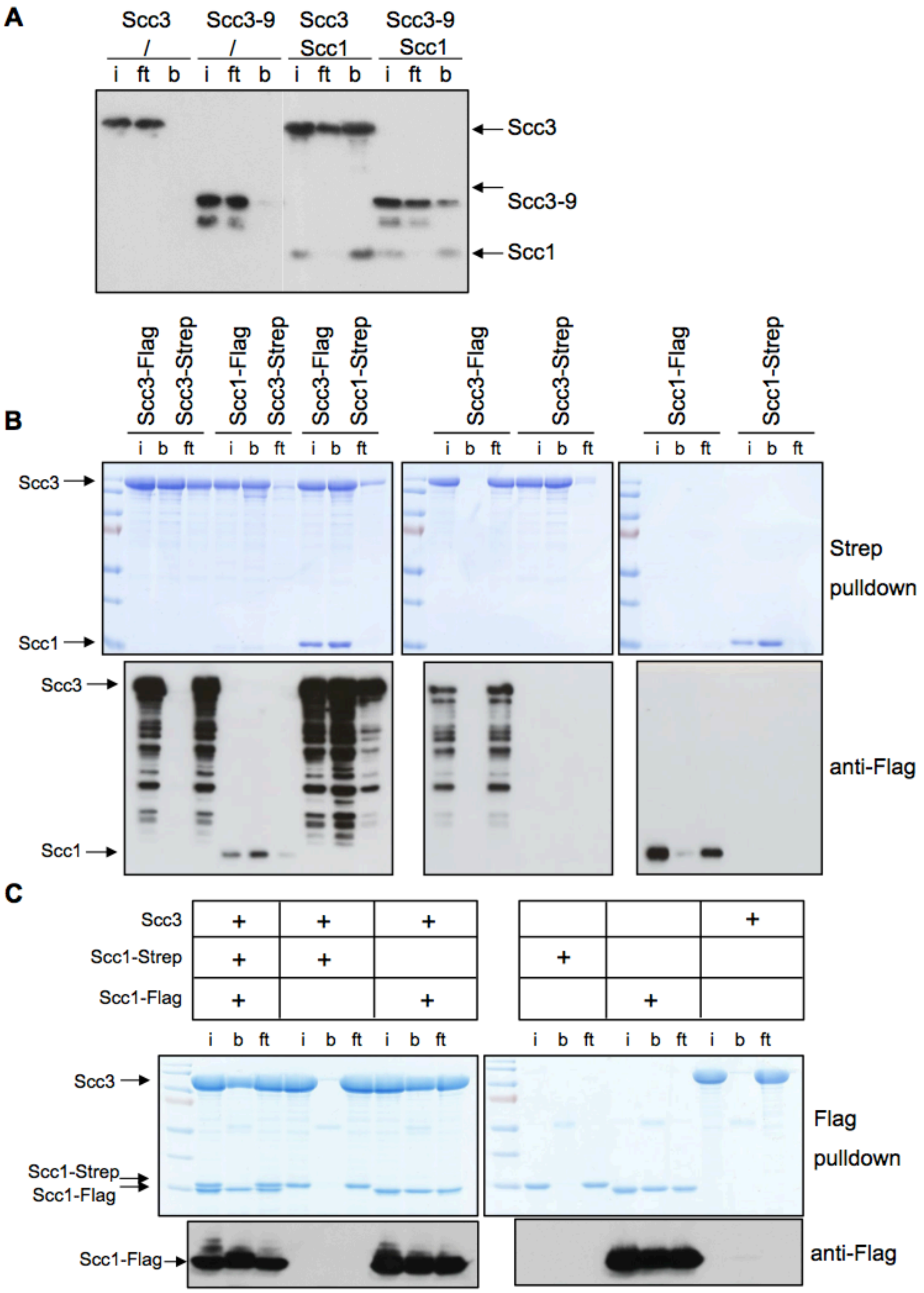


**Figure 2-figure supplement 1. Scc1 forms a 1:1 complex with Scc3's C-terminal domain.**

(A) The C-terminal domain of Scc3 binds to Scc1. Recombinant full length *Sc* Scc3 or the C-terminal fragment (674-1072), Scc3-9, both N-terminal His-tagged, were incubated with an N-terminal His-tagged and C-terminally strep-tagged fragment of

*Sc* Scc1 (259-451) containing the binding sites for Scc3 (Fig. 2). To measure binding of Scc3 to Scc1, samples were subjected to co-immunoprecipitation using Strep-beads, followed by SDS-PAGE and western blot with anti-His antibody (i: input, ft: flow through, b: bound). (B) To test if *Sc* Scc3 forms homodimers, full length versions of the recombinant protein Scc3 either Flag- or Strep-tagged at their C-termini were incubated in an equimolar ratio and proteins immuno-precipitated using Strep-beads. Samples were then subjected to SDS-PAGE and Western blotting using anti-Flag antibody (i: input, ft: flow through, b: bound). A fragment of *Sc* Scc1 fragment (259-451) C-terminal flag-tagged containing the Scc3 binding sites (Fig. 2) was used as a positive control for Scc3 co-immunoprecipitation. (C) To assess stoichiometry of the Scc1/Scc3 complex, two versions of the recombinant protein fragment *Sc* Scc1 (259-451), one Flag-tagged and the other Strep-tagged (at their C-termini), were incubated in a equimolar ratio with *Sc* Scc3. Proteins were then immuno-precipitated using flag-beads and samples run on SDS-PAGE and stained with Coomassie. Western blot using anti-flag antibody was performed to identify the immunoprecipitated *Sc* Scc1 (259-451) C-terminal flag-tagged version.

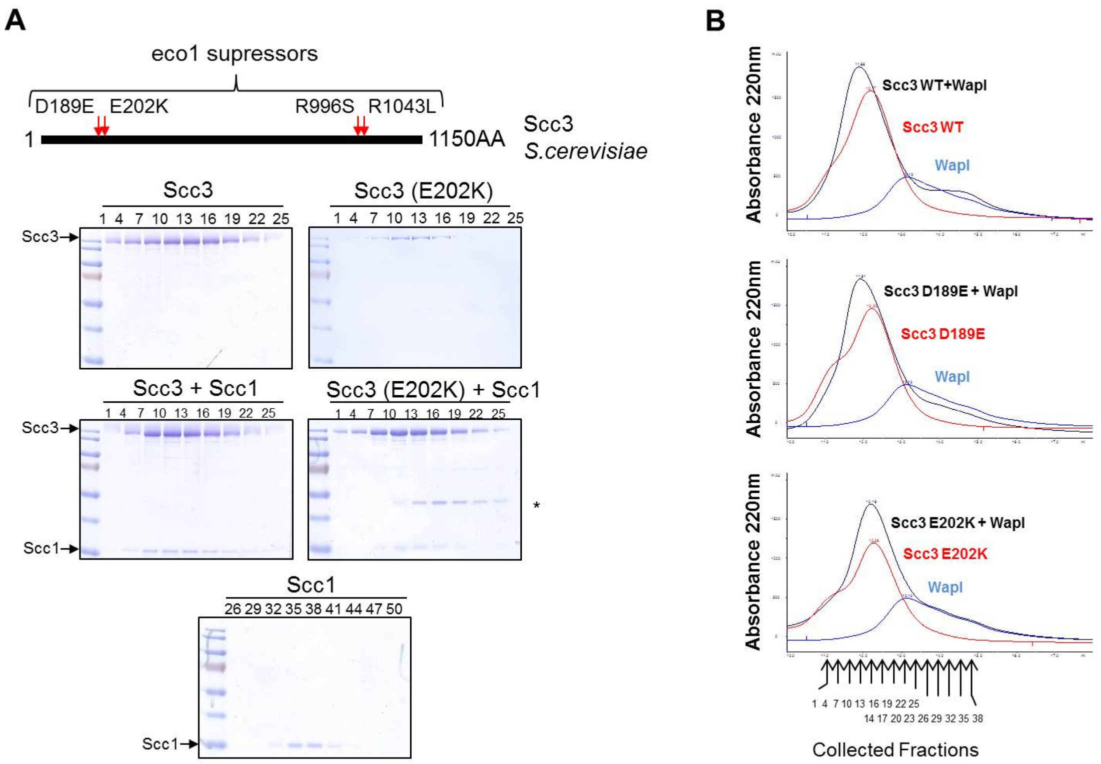


**Figure 3-figure supplement 1. *Sc* Scc3 releasing activity mutant (E202K) binds to Scc1.**

(A) Wild type or mutant (E202K) *Sc* Scc3 protein was incubated either alone or with a fragment of *Sc* Scc1 (259-451) containing Scc3 binding sites (Fig 2). After separation of the proteins by gel filtration, fractions depicted above each lane were analyzed by SDS-PAGE and Coomassie staining. (B) Gel filtration profiles of Scc3 wild type or releasing activity mutants (D189E or E202K) binding to Wapl, as shown in Fig 3.

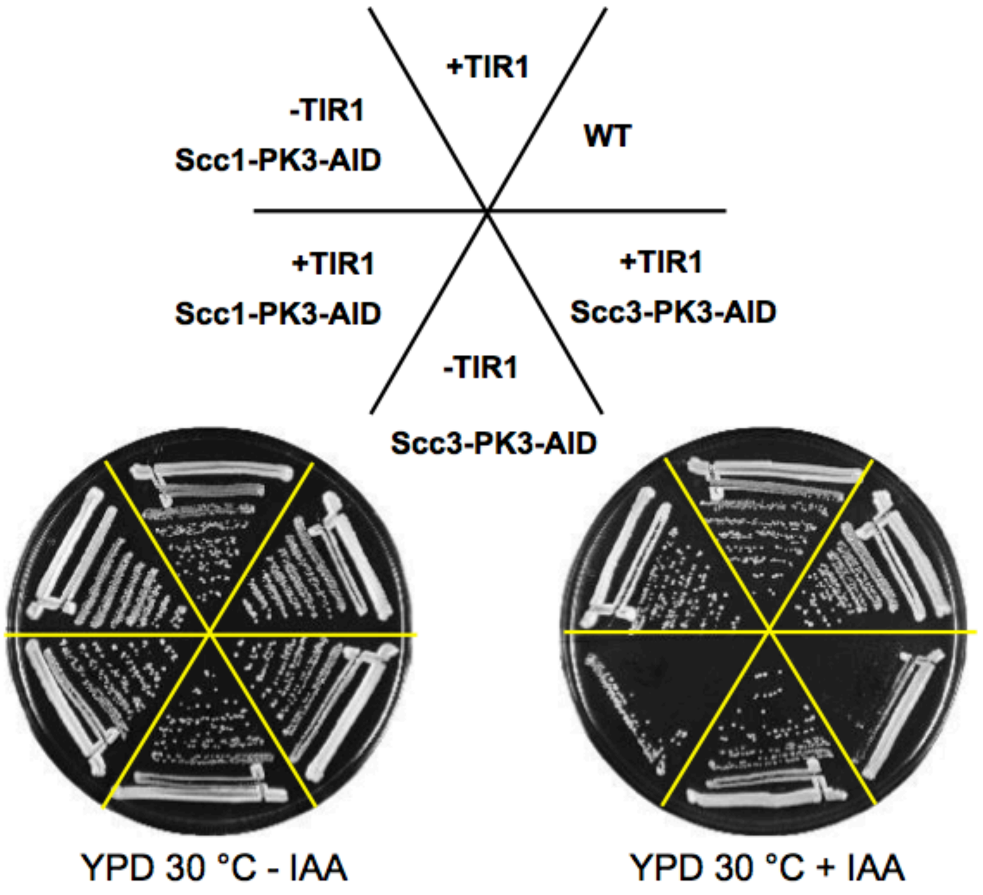


**Figure 4-figure supplement 1. Scc3 auxin-inducible degron (AID).**

Cells whose *SCC3* or *SCC1* genes have been replaced by versions tagged at their C-termini with auxin-inducible degrons (AID) with and without the auxin receptor TIR1 (Nishimura et al., 2009) were streaked to single colonies on YEPD plates lacking or containing auxin (IAA). Auxin prevented proliferation of Scc3-AID expressing cells (K20316) and greatly slowed proliferation of Scc1-AID cells (K18154) but does not alter the growth of cells lacking TIR1 (K20795, K20791), cells expressing just the auxin receptor TIR1 (K17696), or wild type cells (K699).

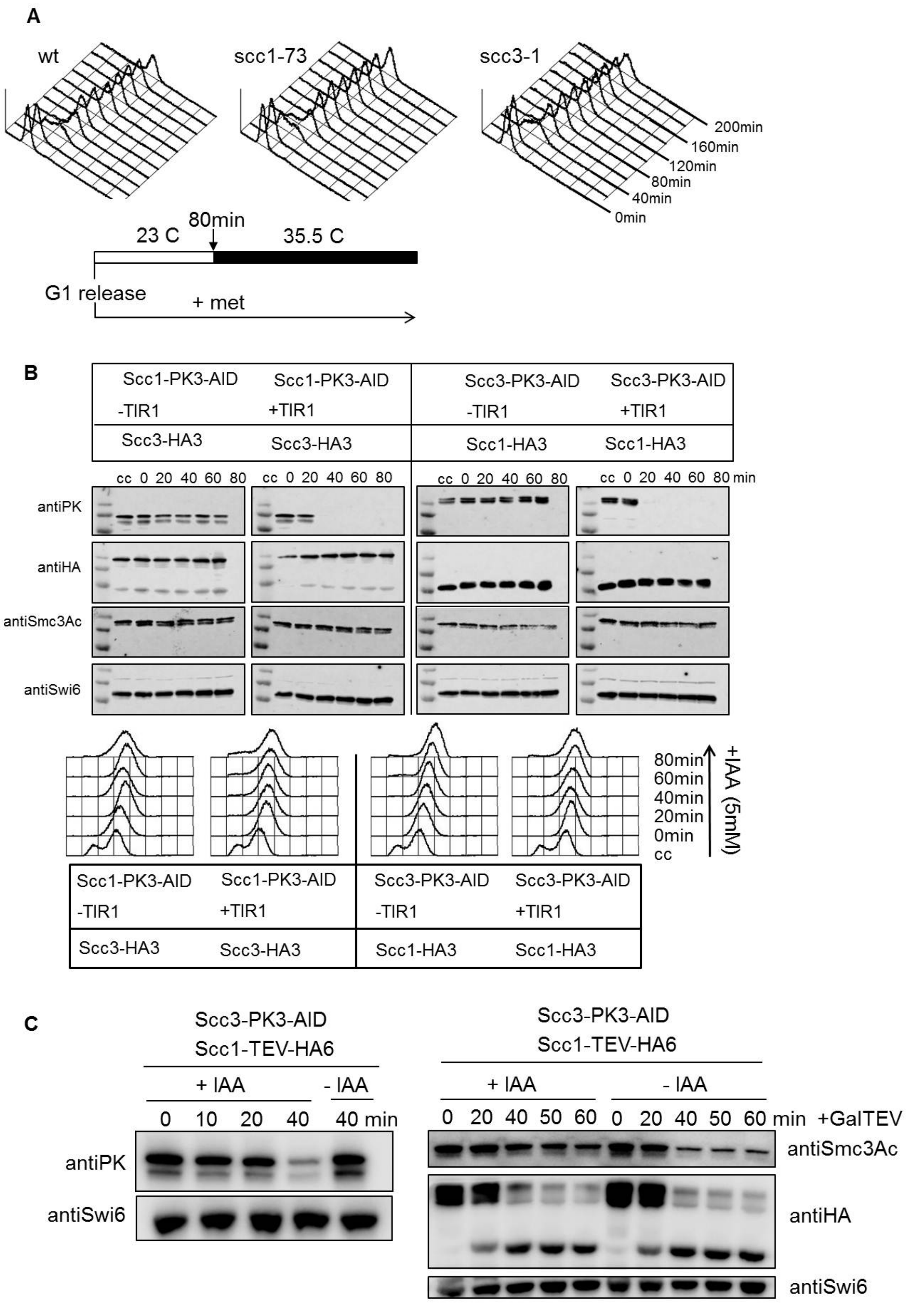


**Figure 4-figure supplement 2. Scc3 maintains cohesion.**

(A) DNA content analysis by FACS of wild type (K15024) or temperature sensitive *scc1-73* (K15031) and *scc3-1* (K15071) cells following release at 23 °C from pheromone-induced G1 arrest in the presence of methionine, which represses *CDC20*

expression. When cells had arrested in metaphase (T = 0 min), cultures were shifted to non-permissive temperature (35 °C) and sister chromatid separation measured by microscopy (Fig 4A). (B) Cells with endogenous Scc1 or Scc3 (K20783) C-terminal tagged with auxin-inducible degron (AID), expressing (K20787, K20783) or not (K20789, K20785) the auxin receptor TIR1, were arrested in G2/M with nocodazole. When arrested in G2/M, auxin (5 mM) was added (T = 0 min) and Scc1, Scc3 and Smc3 acetylation levels were measured at the indicated time points (Fig 4B). G2/M arrest with nocodazole was assessed by DNA content analysis by FACS. (C) As in panel 4B, G2/M arrested cells with nocodazole were depleted of Scc3 in presence of auxin (left panel). After Scc3 depletion (T = 40 min), Scc1 with three inserted TEV sites at residue 220 was cleaved upon TEV protease induction by its Gal promoter (KN23050). Scc1TEV cleavage and Smc3 acetylation levels were measured at the indicated time points.

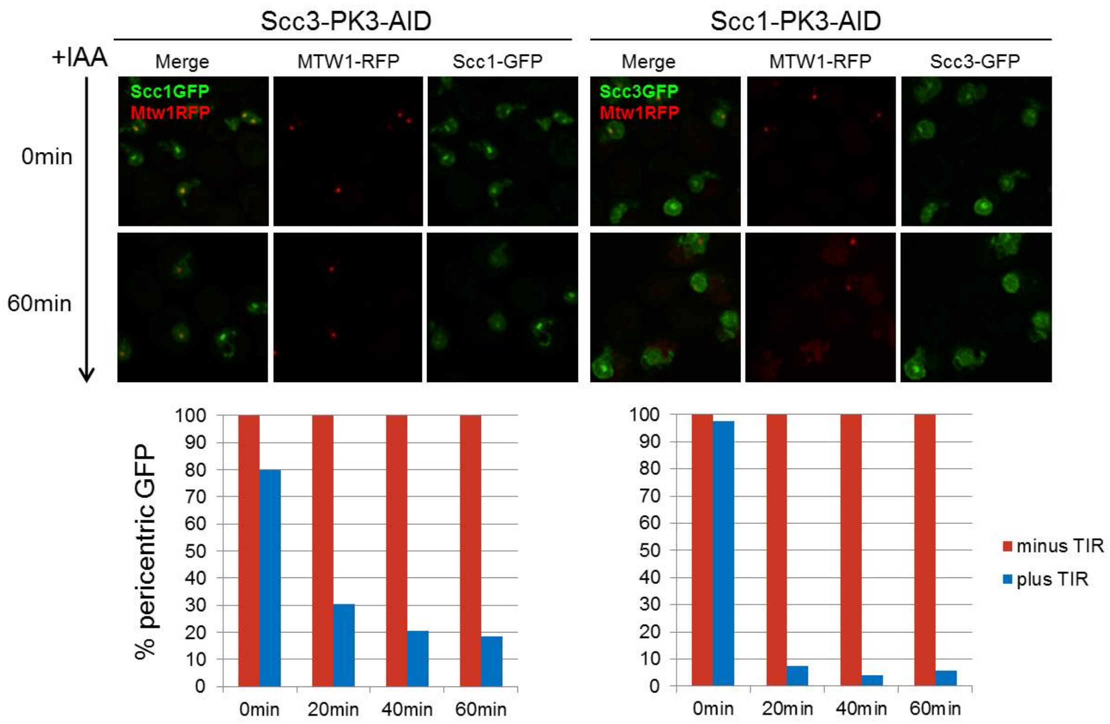


**Figure 4-figure supplement 3. Degradation of *Sc* Scc3 causes a loss of cohesin structure.**

Live cell imaging showed peri-centric Scc1 or Scc3 tagged at their C-termini with GFP in G2/M cells arrested in nocodazole (T = 0 min). Degradation of Scc3 (K20854) or Scc1 (K23181) by the auxin-inducible degron caused a loss of peri-centric cohesin structures and an increase of GFP signals in the nucleoplasm after 20 min when observing Scc1-GFP or Scc3-GFP respectively, while peri-centric cohesin is unaltered in the absence of the ligase TIR1 (K23382, K23385). Centromeres were labeled with Mtw1-RFP only on the cells expressing the TIR ligase (plus TIR) (KN20854, KN23181), but not on the control cells (minus TIR) (KN23382, KN23385). Both populations (minus/plus TIR) where mixed for each respective degron for a further quantification of peri-centric cohesin (GFP signal) on the same slide.

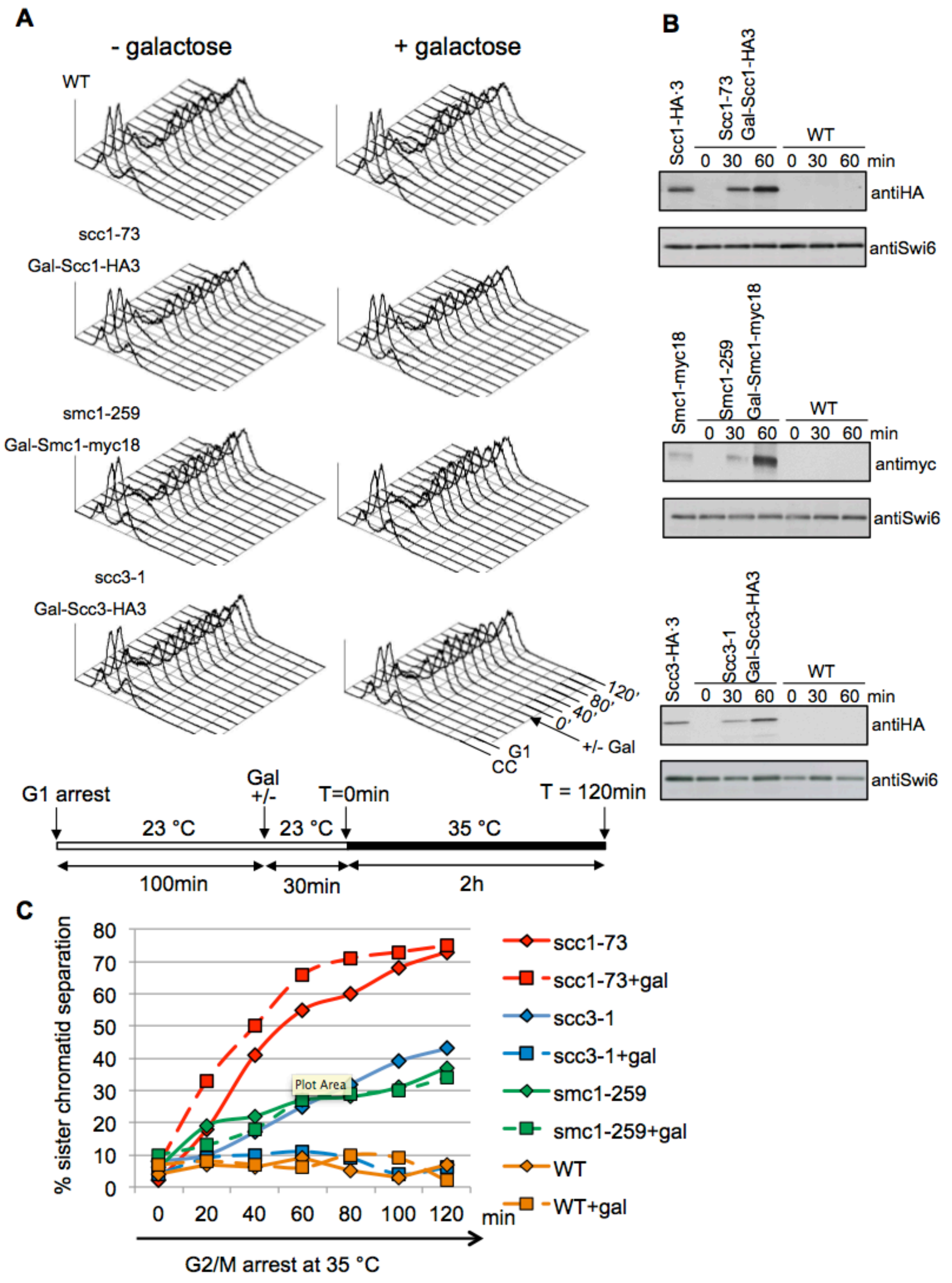


**Figure 4-figure supplement 4. Scc3 turnover during G2/M.**

(A) DNA content analysis by FACS of cells expressing the untagged endogenous temperature sensitive mutant alleles *scc1-73* (K16680), *smc1-259* (K16679) or *scc3-1* (K16678), and their corresponding wild type proteins under the *GAL1-10* promoter. Cells were released from α-factor induced G1 arrest at 23 °C into YEPRaff medium containing methionine, which repressed *CDC20* expression and caused metaphase

arrest. After 100 min., galactose was added to induce wild type version of the proteins and the cultures shifted to the restrictive temperature (35 °C) 30 min later (Fig 4D). (B) Protein levels were measured after galactose induction in metaphase arrested cells at the indicated time points and compared to levels made by endogenous tagged versions of each gene (K16677, K8266, K12568, K7606). (C) The percentage of cells with double (split) GFP dots at the *URA3* locus was measured by microscopy following the temperature shift to the non permissive temperature 35 °C (T = 0 min) (Fig 4D).

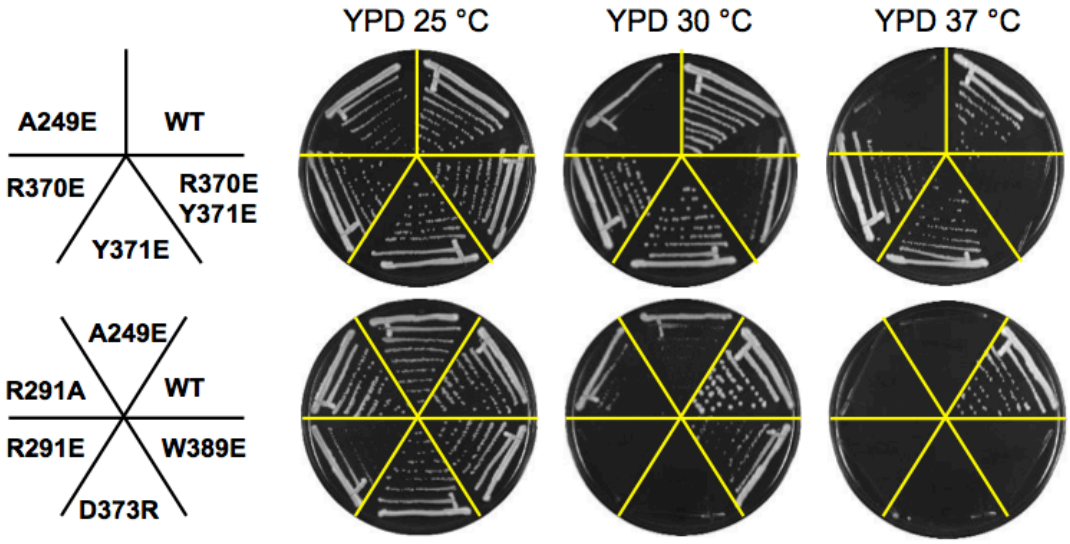


**Figure 5-figure supplement 1. Scc3 CES temperature sensitive alleles.**

Wild type (K21817) and mutant cells (K23026, K21329, K23289, K21443, K21719, K23235, K21713) streaked on YEPD plates incubated at different temperatures. All but one strain lacked the endogenous *SCC3* gene, the locus of which was marked by a Nat resistance marker, and carried either wild type or mutant *SCC3* genes integrated at the *leu2* locus. Scc3A249E (K23048) was expressed from the *SCC3* locus (original *scc3-1* allele) (Toth et al., 1999).

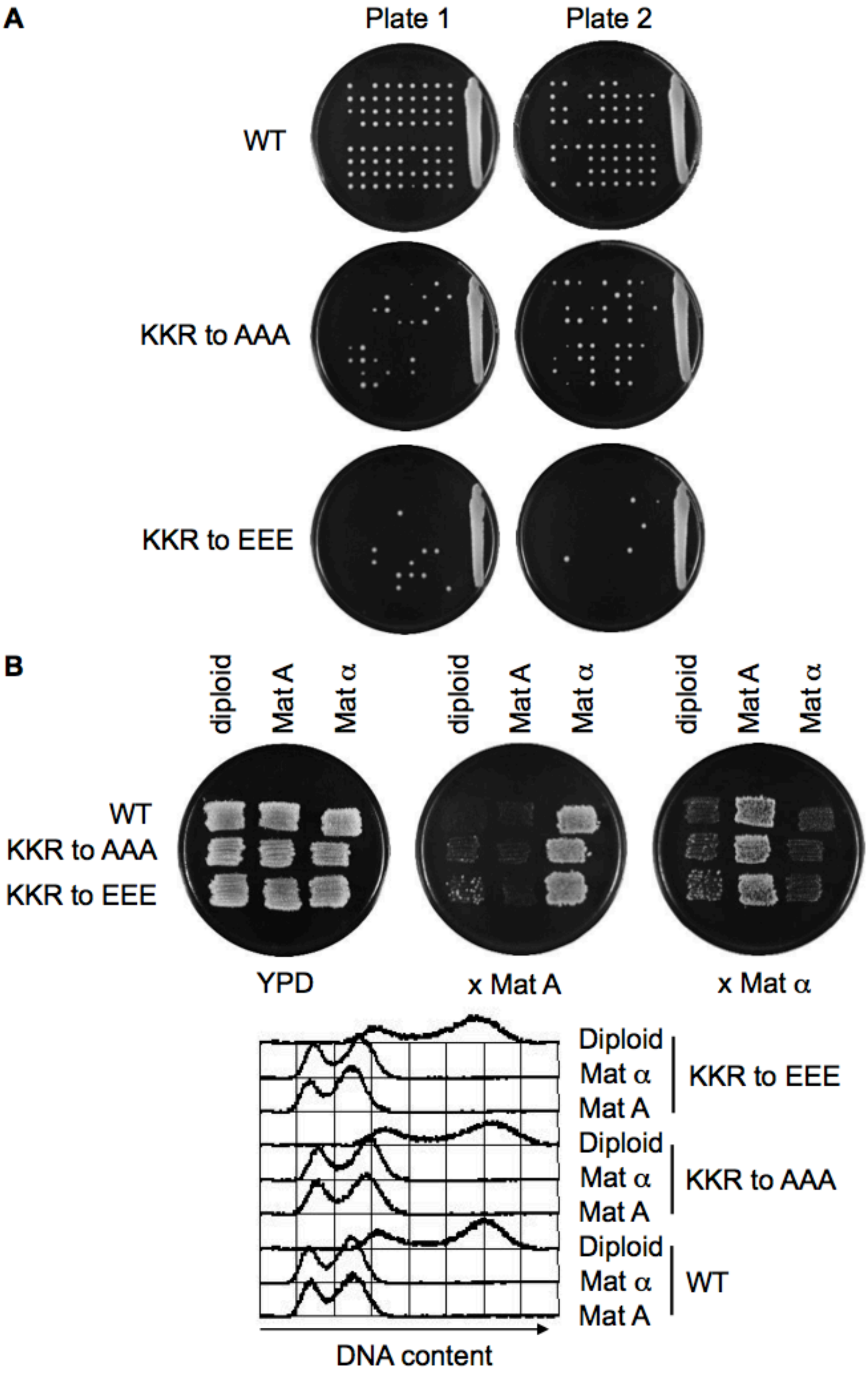
A
Plate 1
Plate 2
WT
KKR to AAA
KKR to EEE
B
diploid
Mat A
Mat α
diploid
Mat A
Mat α
diploid
Mat A
Mat α
WT
KKR to AAA
KKR to EEE
YPD
x Mat A
x Mat α
Diploid
Mat α
Mat A
KKR to EEE
Diploid
Mat α
Mat A
KKR to AAA
Diploid
Mat α
Mat A
WT
DNA content

**Figure 5-figure supplement 2. Tetrad dissection of K372A/E K404A/E R449A/E triple mutants**.

(A) Homozygous diploids in which either wild type (K21815) or mutant *SCC3* genes (K23276, K23279) integrated at the *leu2* locus (endogenous *SCC3* locus deleted) were sporulated on plates and four-spored asci dissected (18 tetrads/plate). Note that because both triple mutations reduced the frequency of four-spored asci, tetrad dissection of those produced will under-estimate the effect on the production of viable spores. (B) Mating tests demonstrating non-mating behavior of diploid and FACS analysis showing 2N DNA contents of diploid strains, when compared to their parental haploid strains (K21817, K21818, K23277, K23278, K23280, K23281).

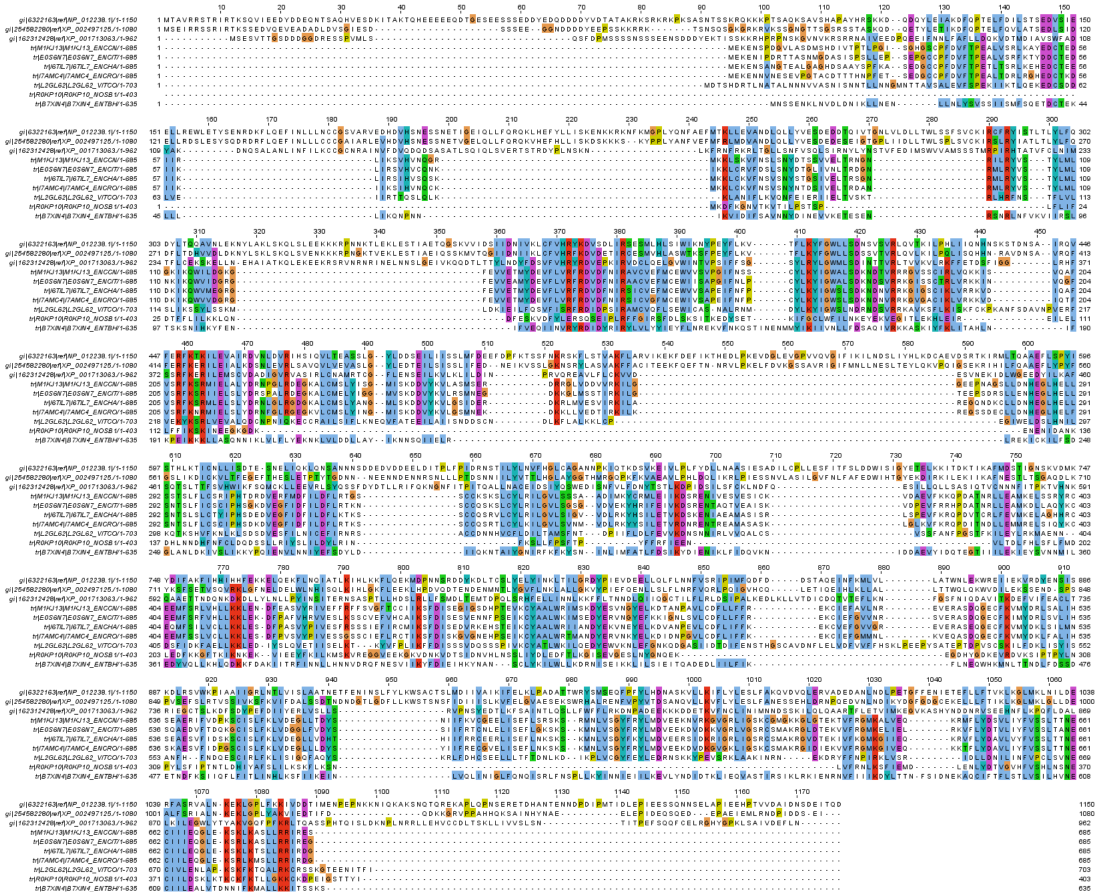

**Figure 5-figure supplement 3. Alignment of microsporidian Scc3 orthologs with their yeast counterparts.** The following Scc3 sequences were included: *Saccharomyces cerevisiae* (P40541), *Zygosaccharomyces rouxii* (XP_002497125.1), *Encephalitozoon cuniculi* (M1KJ13), *Encephalitozoon intestinalis* (E0S6N7), *Encephalitozoon hellem* (I6TIL7), *Encephalitozoon romaleae* (I7AMC4), *Vittaforma corneae* (L2GL62), *Nosema bombycis* (R0KP10), *Enterocytozoon bieneusi* (B7XIN4), *Edhazardia aedis* (J9DKH5).
